# Sociocognitive Functioning and Psychosocial Burden in Patients with Brain Tumors

**DOI:** 10.3390/cancers14030767

**Published:** 2022-02-01

**Authors:** Milena Pertz, Uwe Schlegel, Patrizia Thoma

**Affiliations:** 1 Department of Neurology, University Hospital Knappschaftskrankenhaus, Ruhr University Bochum, In der Schornau 23–25, D-44892 Bochum, Germany; uwe.schlegel@kk-bochum.de; 2 Neuropsychological Therapy Centre (NTC), Faculty of Psychology, Ruhr University Bochum, Universitätsstraße 150, D-44780 Bochum, Germany; patrizia.thoma@rub.de

**Keywords:** brain tumor, quality of life, psychosocial burden, social cognition, sociocognitive functioning

## Abstract

**Simple Summary:**

After years of gauging the efficacy of tumor-directed therapies primarily by means of survival, a broader perspective on therapeutic outcome also focusses on patients’ everyday functional abilities. Besides neurocognition, a matter of high clinical relevance, “social cognition” may also affect well-being and quality of life (QoL) in brain tumor patients. Abilities that enable individuals to establish and maintain social relationships are summarized under the umbrella term “sociocognitive functioning”. These abilities encompass the understanding and sharing of emotional and mental states of other individuals as well as skills to detect and resolve interpersonal problems. These sociocognitive abilities may be challenged in highly demanding life situations such as brain tumor diagnosis and treatment. Therefore, we summarize the literature on psychosocial burden and sociocognitive functioning in adult brain tumor patients.

**Abstract:**

Brain tumors may represent devastating diseases and neuro-oncological research in the past solely focused on development of better treatments to achieve disease control. The efficacy of tumor-directed treatment was evaluated by progression-free and overall survival. However, as neuro-oncological treatment became more effective, preservation and improvement of quality of life (QoL) was noticed to represent an important additional outcome measure. The need to balance between aggressive tumor-directed treatment and preservation of QoL was increasingly acknowledged in brain tumor patients. QoL is comprised by many determinants; one of those may have been rather neglected so far: social cognition. Since diagnosis and treatment of brain tumors represent demanding life situations, patients may experience increased psychosocial burden and the negative consequences of illness on well-being may be buffered by intact social relationships. These skills to build and maintain supportive social relationships essentially depend on the ability to empathize with others and to recognize and appropriately address social conflicts, i.e., “sociocognitive functioning”. Therefore, sociocognitive functions may influence QoL and treatment outcome. In this article, we review the literature on psychosocial burden and sociocognitive functioning in adult brain tumor patients.

## 1. Introduction

Brain tumors represent devastating diseases and for decades of neuro-oncological research the majority of studies has concentrated on mere patient survival. For instance, when evaluating tumor-directed therapies in clinical trials progression-free survival (PFS) and overall survival (OS) were deemed as unique endpoints of treatment efficacy. Due to more effective tumor-directed therapies survival rates increased in a substantial fraction of brain tumor patients [1,2]. This for instance applies to medulloblastoma [3], primary central nervous system lymphoma (PCNSL) [4] as well as to subgroups of gliomas [5,6]. Thus, long-term sequelae of the disease and/or consequences of treatment became more relevant [7]. To this end, survival alone is no longer considered as an adequate outcome measure when evaluated in an isolated manner [8,9]. Patients’ “quality of survivorship” may be diminished by impairments of daily functioning in response to (residual) tumor and/or aggressive tumor-directed treatment. Therefore, the possible benefits of intensive tumor-directed treatment to achieve disease control ought to be outweighed against the risks of functional deficits, neurological impairment and treatment-related neurotoxicity [10,11,12]. This is particularly important for those brain tumor patients [4,8,13,14] who have a long life expectancy and can survive in a stable state for years. However, this also applies to patients with highly malignant brain tumors, in whom not only prolongation of survival but also preservation of quality of life (QoL) as long as possible in the remaining lifetime is aspired. Consequently, the maintenance of QoL has been contemplated in neuro-oncological studies as a surrogate of efficacy and tolerability of tumor-directed treatment in the last years.

When considering the patients’ QoL, neurocognitive functioning plays a major role. Even mild cognitive deficits can detrimentally affect a person’s abilities to perform daily activities as well as social and occupational roles, to maintain interpersonal relationships and leisure activities. Based on this, the possible cognitive side- and long-term effects of brain tumors and tumor-directed treatments were focused intensively in the last years of neuro-oncological research [6,14,15,16,17,18,19,20,21,22,23,24,25,26,27,28,29,30,31,32,33,34,35,36,37,38,39]. The incidence of neurocognitive impairment varies from 12.5% to 91% [40,41,42,43,44,45,46]. However, this variability might not be solely attributed to heterogeneous study methodology. Cognitive functioning and its deterioration may be affected by the tumor itself (i.e., biological factors), its treatment (surgery, radiotherapy, chemotherapy or its combination), i.e., medical factors, the use of associated medication (e.g., antidepressants, antiepileptic medication), as well as psychological factors such as mood and/or fatigue.

In addition to suffering from neurocognitive disturbances, brain tumor patients are simultaneously burdened by symptoms of oncological diseases (e.g., uncertain prognosis and fear of disease progression) and by neurological symptoms (e.g., focal symptoms as paresis, aphasia, visual field defects and personality changes) [47]. Brain tumor patients endure the threat to their lives and to their sense of self [47] that may lead to combined neuro-oncologic specific fears and distress symptoms [48], i.e., “double threat”.

Due to the functional, cognitive and/or emotional disturbances of the disease as well as due to the social stigma of suffering from a brain tumor, brain tumor patients experience psychosocial burden and distress [49,50]. According to the National Comprehensive Cancer Network (NCCN) psychosocial distress is defined “as a multifactorial, unpleasant experience of a psychological (i.e., cognitive, behavioral, emotional), social, spiritual and/or physical nature that may interfere with the ability to cope effectively with cancer, its physical symptoms and its treatment” [51] (p. 2). Brain tumor patients face many life changes, such as loss of independence due to potential physical restrictions, problems with resuming work and decreases in cognitive functioning. Furthermore, changes in personality, feelings of social isolation and changes of interpersonal dynamics may occur. These aspects may lead to a changing of roles with the family, friends and other caregivers as well as difficulties with social relationships [52]. Vice versa, supportive social relationships may prevent a patient from pathological distress caused by the illness and its treatment and thus may positively influence QoL and health outcomes [53,54,55,56,57,58]. Models targeting adult patients with acquired brain injuries of various etiologies proposed a number of factors contributing to psychosocial functioning. For instance, impaired cognitive functioning (in particular executive functioning), the severity of the injury, the occurrence of mood disorders and psychological stress as well as timing of the injury and duration of the recovery process may represent factors associated with poor psychosocial functioning [59,60]. By contrast, distinct personal resources (such as positive problem orientation, coping resources, behavioral and emotional regulation, meta-cognitive abilities, self-awareness and internal locus of control) as well as environmental resources (such as beneficial sociocultural context and positive past experiences as well as social support and functioning of the immediate family) may positively influence psychosocial functioning of patients [60,61]. In this vein, social well-being is defined as the ability of patients to engage in their social network and usual lifestyle [62]. 

Abilities that facilitate adequate social behavior and maintaining of social relationships are known under the umbrella term “social cognition” or “sociocognitive functioning” [63,64]. While psychosocial burden, as defined earlier, is related to the patients themselves and represents the burden experienced as a consequence of the disease and its treatment, sociocognitive functioning is a more performance-based construct gauging the ability to understand, empathize with and appropriately interact with other people. Some interdependence between the constructs may be assumed such that increased psychosocial burden may limit the ability to focus on the mental and emotional states of others. Vice versa, impaired sociocognitive functioning and ensuing conflicts may contribute to psychosocial burden. Social cognition encompasses different but interrelated psychological constructs that range from more elementary functions, such as emotion recognition, to more complex ones. Emotion recognition is the ability to identify human emotional states based on facial or vocal cues [65]. More complex or higher-order sociocognitive functions include concern for others (empathy), perspective taking of others’ mental states (Theory of Mind, ToM) and social problem solving. The individual’s understanding of and emotional response to the observed or imagined emotional experience of another person is denoted by the term empathy [66]. Most of the studies postulate a subdivision into cognitive and affective empathic facets [67]. These facets indicate the ability to affectively share another person’s emotional state (emotional empathy) and to cognitively understand another person’s feelings (cognitive empathy) [68]. Cognitive empathy conceptually coincides with affective ToM that implies the ability to understand and infer the emotions of others on a cognitive level [68,69]. Social problem solving embodies one of the most complex sociocognitive abilities. It encompasses the identification of an interpersonal conflict and the production and selection of appropriate and effective strategies to resolve such a conflict [70,71]. Sociocognitive functions enable humans to comprehend other peoples’ behavior in the context of a specific situation by understanding what is going on in other peoples’ minds and based on the latter, to adapt their own behavior in social situations in a goal-directed manner. The capability to take the perspective of another persons’ emotions and intentions is needed in virtually all human interactions and thus offers an important basis for a person’s social relationships and group membership. Therefore, not only neurocognitive functioning but also sociocognitive functioning is an essential part of daily human life.

Acknowledging the fact that brain tumor patients may benefit from supportive social relationships during the disease course, the aim of this review is to provide a systematic overview of the available data whether and how sociocognitive functioning is altered in adult brain tumors patients. Since social group membership is actually an essential factor in maintaining the well-being of a healthy individual [72,73], it is obvious that patients with severe or life-threatening illnesses are even more dependent on support from caregivers and their social group. Furthermore, the review will focus on the extent to which those patients experience psychosocial burden in response to the tumor and its treatment to shed light on consequences of the brain tumor and its treatment on psychosocial well-being. Both, studies on primary and secondary brain tumor patients will be included in the present review. An earlier review on social cognition in patients with intracranial tumors [74] reviewed the literature from the angle of cognitive neuroscience (i.e., neuroimaging results and neuroanatomical correlates of social cognition) and a bibliometric analysis has demonstrated the increasing interest for concepts such as psychosocial burden and sociocognitive impairment in recent years [75]. Furthermore, recently the relevance of brain mapping on social cognition and underlying white matter fiber tracts was summarized by Nakajima et al. (2021) [76]. To this end, the present review aims to primarily highlight the clinical relevance of sociocognitive impairment in brain tumor patients, to give an overview on behavioral results of the studies and to incorporate most recent publications on both sociocognitive functioning and psychosocial burden in adult brain tumor patients. Better insights in the issues of social cognition and psychosocial burden in brain tumor patients and its clinical relevance may inspire research as well as facilitate the investigation and implementation of supportive sociocognitive treatment interventions in clinical practice. Those developments may positively impact the QoL of both brain tumor patients and their caregivers.

## 2. Methods

### 2.1. Search Strategy

To identify relevant articles a literature search was performed in the electronic data bases of PubMed and Web of Science.

The following search string was used in PubMed data bases: ((“social cognition” [Title]) OR (“sociocognitive*” [Title]) OR (“theory of mind” [Title]) OR (“mentaliz*” [Title]) OR (“empath*” [Title]) OR (“emotion recognition” [Title]) OR (“social problem solving” [Title]) OR (“social skill*” [Title]) OR (“social funct*” [Title]) OR (“social impairment*” [Title]) OR (“psychosocial impairment*” [Title]) OR (“psychosocial*” [Title]) OR (“psychosocial burden*” [Title]) OR (“psychosocial difficult*” [Title]) OR (“social support” [Title])) AND ((“brain tumour*” [Title]) OR (“brain tumor*” [Title]) OR (“brain neoplasm*” [Title]) OR (“intracranial neoplasm*” [Title]) OR (“brain cancer*” [Title]) OR (“intracranial tumour*” [Title]) OR (“intracranial tumor*” [Title]) OR (“glioma*” [Title]) OR (“low-grade glioma*” [Title]) OR (“low-grade tumour*” [Title]) OR (“low-grade tumor*” [Title]) OR (“low-grade*” [Title]) OR (“high-grade glioma*” [Title]) OR (“high-grade tumour*” [Title]) OR (“high-grade tumor*” [Title]) OR (“high-grade*” [Title]) OR (“meningioma*” [Title]) OR (“primary central nervous system lymphoma*” [Title]) OR (“brain metastas*” [Title])) NOT ((“child*” [Title]) OR (“paediat*” [Title]) OR (“pediat*” [Title])).

To identify relevant literature in the Web of Science core collection data bases the following search string was used: TI = ((social cognition OR sociocognitive* OR theory of mind OR mentaliz* OR empath* OR emotion recognition OR social problem solving OR social skill* OR social funct* OR social impairment* OR psychosocial impairment* OR psychosocial* OR psychosocial burden* OR psychosocial difficult* OR social support) AND (brain tumour* OR brain tumor* OR brain neoplasm* OR intracranial neoplasm* OR brain cancer* OR intracranial tumour* OR intracranial tumor* OR glioma* OR low-grade glioma* OR low-grade tumour* OR low-grade tumor* OR low-grade* OR high-grade glioma* OR high-grade tumour* OR high-grade tumor* OR high-grade* OR meningioma* OR primary central nervous system lymphoma* OR brain metastas*)) NOT TI = (child* OR paediat* OR pediat*).

The literature included original articles from April 1986 to August 2021 as identified by the literature search as well as original articles as found by manual searches and screening of the references (i.e., additional articles were identified through cross-referencing of the retrieved articles). The literature search took place on 22 April and was repeated on 22 August 2021 to screen for timeliness of data. 

### 2.2. Selection Criteria

Articles included were original peer-reviewed articles reporting sociocognitive functioning and/or psychosocial burden in adult (≥18 years) brain tumor patients. All types of studies were considered as long as they presented original research findings. Since different definitions of social cognition/sociocognitive functioning and psychosocial burden were presented in the literature the included articles had to cover at least one of the different constructs as mentioned above (see Section 1 and search string in Section 2.1). Sociocognitive impairment and psychosocial burden could have been reported from the patients’ or the caregivers’ perspective but had to be objectively assessed in the patients. Studies only reporting psychosocial burden in caregivers of brain tumor patients were excluded. Furthermore, if the studies focused on psychiatric comorbidities only or used the distress thermometer as the only screening tool, they were excluded. The distress thermometer represents a single item screening tool for distress using a visual analogue scale on which participants rate their level of distress from 0 (none) to 100 (extreme). Although the distress experienced in brain tumor patients is reported to be high [77] the present review aimed to specifically target psychosocial burden. Therefore, studies assessing distress alone go beyond the scope of this review. The same is true for studies which focused on psycho-oncological and psychosocial support only. Further exclusion criteria were as follows: articles not written in English, studies involving only children (<18 years), studies including both adults and children but without a subanalysis involving only adults and studies of entities other than brain tumors. Furthermore, meeting and conference abstracts were excluded from the analyses. Additionally, (systematic) reviews that did not include original data as well as study protocols, letters to the editor and editorial material that did not include original results and comments/notes were excluded from the present review. 

### 2.3. Data Extraction

From all articles the following data was extracted manually: authors, year of publication, study design, time of assessment (e.g., pre- or post-treatment), number of patients with their diagnosis (if explicitly reported), presence of a clinical or healthy control group, criteria for matching with the control group (if present), instruments to assess sociocognitive functioning or psychosocial burden and key findings or research objective concerning sociocognitive functioning or psychosocial burden. The key findings were classified as comparison of (mean) scores between groups at one time point, association of sociocognitive functioning or psychosocial burden with other outcomes, sociocognitive functioning or psychosocial burden as an outcome or a predictor in a prognostic model, comparison of (mean) scores in one group over time or comparison of (mean) scores between groups at multiple time points. 

## 3. Results 

### 3.1. Genereral Search Results 

**Figure 1:**
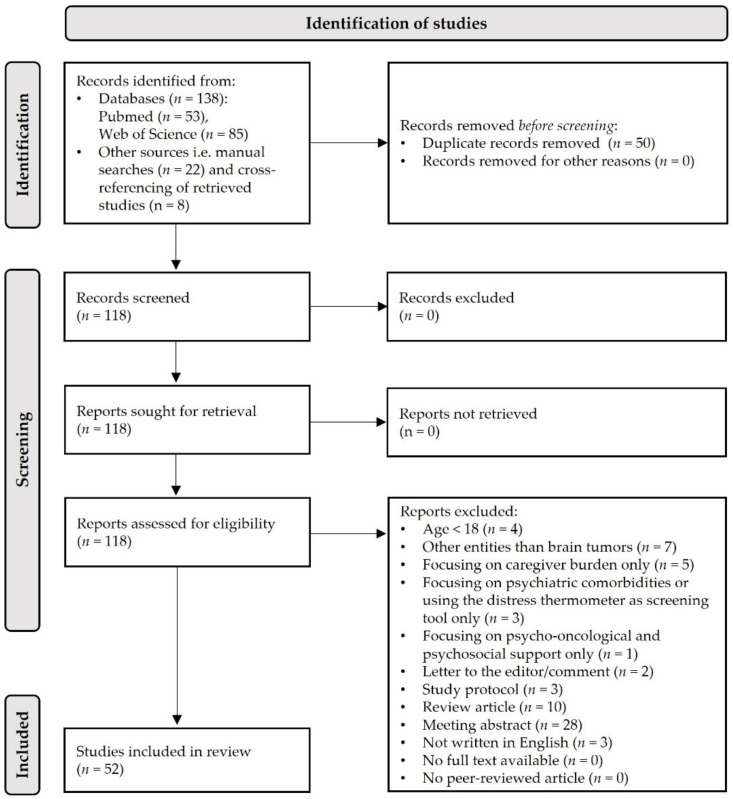
Flow diagram on searches of PubMed and Web of Science databases and studies included in the review.

The literature search resulted in 138 records as identified by PubMed and Web of Science searches using the search strings specified above. Another 22 studies were identified by manual searches and eight additional studies were identified by screening the references of the retrieved literature for relevant articles. Overall, 168 records were retrieved. After removing 50 duplicates, 118 reports were screened manually. Sixty-six articles were excluded (see exclusion criteria of the present review in Section 2.2). Finally, 52 studies were eligible and included in the review (see Figure 1 for the article selection procedure according to PRISMA guidelines [78]). 

### 3.2. Psychosocial Burden 

Of the 52 studies, 20 assessed psychosocial burden in brain tumor patients. Of these, one study included preoperative patients and 13 studies addressed psychosocial burden and the relevance of social relationships in brain tumor patients after treatment. Another six studies used more than one assessment during the disease course. See Table 1 for the study design and main instruments to assess psychosocial burden and Table S1 for a summary of the study methodology and detailed descriptions of the main results. 

In general, psychosocial burden was apparent in brain tumor patients before any treatment has started [94]. Furthermore, both immediately after diagnosis or in the early treatment phase [92,93,104] as well as during the course of the disease (i.e., also months or years later) [83,126] psychosocial burden appears to be moderate to high. Common challenges were problems of dealing with the partner or children [93], role reversals, strain and concerns about the impact of illness on caregivers [104]. Relevant psychosocial stress was found in 73% of brain tumor patients immediately after surgery [92]. Since psychosocial burden may be caused by the brain tumor diagnosis and the distress associated with the surgical treatment [92] social support might be particularly important in the early treatment phase. However, other studies highlighted the relevance of social support at different states of the disease [124]. In this vein, in general the prevalence of psychosocial needs was high in the outpatient setting [83,106,126] highlighting the relevance of supportive social relationships when the disease continues to progress. By contrast, patients with benign tumors reported their perceived social support as high several years after diagnosis [102]. Some studies identified treatment specific and illness-inherent factors (malignancy and occurrence of seizures) influencing the severity of psychosocial burden [79,103,121,128], other studies generally showed an increased psychosocial burden [83,106,126]. Some studies with more than one timepoint pointed to a lower psychosocial burden during the disease course [105] while other studies reported an increase of psychosocial burden during the course of the disease [86,89]. Some patients emphasized positive effects on their relationships while others described a loss of relationships due to the tumor and its treatment [113]. In a recent study, the most consistent predictor of QoL was the number of social contacts when assessed weekly over a period of 12 weeks in brain tumor patients during the first lockdown of the COVID pandemic [125]. This study highlighted the impact of social group membership on well-being in brain tumor patients which probably is also valid independently of the COVID pandemic [90]. Additionally, some studies demonstrated a positive influence of psychotherapeutic interventions targeting psychosocial issues [114] and indicated the positive influence of supportive social relationships on the well-being of brain tumor patients in general [90].

### 3.3. Sociocognitive Functioning

While the previous paragraphs demonstrated that brain tumor patients suffer from relevant psychosocial burden and, as a result, the need for social support throughout the disease course, the following sections summarize evidence on sociocognitive functioning in brain tumor patients relevant for the establishment and maintenance of supportive social networks. Of the 52 studies identified in the present review, 33 assessed sociocognitive functioning in brain tumor patients. Of these, five studies assessed sociocognitive functioning prior to treatment, 15 studies focused on the posttreatment phase and 13 studies involved multiple assessments during the disease course. See Table 1 for the study design and main instruments to assess social cognition and Table S1 for a summary of the study methodology and a detailed description of the main results.

The studies on sociocognitive functioning in brain tumor patients present contradictory results. For instance, some pretreatment studies report unimpaired performance or only minor sociocognitive deficits in preoperative patients, when addressing crossmodal emotion recognition [107] and cognitive empathy [100] with the latter being assessed with the Reading the Mind in the Eyes Test (RMET). In the RMET, participants had to infer complex mental states (i.e., “embarrassed”) from a person’s eye gaze. Addressing various aspects of sociocognitive functioning such as ToM [94], cognitive and affective empathy, perception of others’ pain and emotional perspective taking [88] as well as emotion recognition [109] other studies pointed to significant sociocognitive impairments even before any treatment has started [88,94,109]. This probably argues for an effect of tumor mass or an impaired functional connectivity due to the localization of the tumor, leading to sociocognitive impairment. Though carried out in a heterogeneous patient group, one of the preoperative studies for the first time highlighted the clinical relevance of sociocognitive impairments in brain tumor patients [94].

The postoperative and posttreatment studies presented contradictory results too. Some of these studies revealed sociocognitive impairments after treatment in single cases or case series [80,81,82,95], some of them region-specific [95]. By contrast, another case study reported normal abilities in various sociocognitive tasks [120]. However, the intact sociocognitive functioning in the laboratory tasks of the latter study stood in sharp contrast to the profoundly impaired social decision-making the patient exhibits in real life [120]. Studies including larger patient groups that focused on the early treatment phase demonstrated impairments in categorial and dimensional emotional decoding in a heterogeneous group of brain tumor patients [116,117]. Furthermore, another study reported impairments in comprehension of mentalistic material especially in brain tumor patients with frontal lesions [87]. Additionally, studies with longer periods between diagnosis/treatment and data assessment presented impairments of emotion recognition in brain tumor patients [123] and supported the notion of a region-specific impairment [101]. In one of these studies, a ventromedial prefrontal brain tumor patient group scored significantly lower on facial emotion recognition. Furthermore, both a ventromedial and dorsolateral prefrontal brain tumor group performed worse concerning ToM [101]. Three studies targeted rather large and homogeneous groups of patients with low-grade glioma after surgery [98,99,111]. These studies reported rather minor to moderate sociocognitive impairments concerning self-reported empathy [99], cognitive empathy (RMET) [98,111] and ToM assessed with a Comic Strip Task [98]. In this task participants had to select the most logical ending of a comic strip among distracters by inferring the intentions of characters. Studies targeting PCNSL reported some conflicting results. While in an early study the self-reported sociocognitive abilities and stress coping abilities were comparable between PCNSL and the normal population [96] a recent study demonstrated impairments in cognitive empathy and social problem solving abilities in PCNSL patients [118]. These contradictory results may be due to an inappropriate targeting of sociocognitive impairment by self-report measures, also affected by potentially reduced metacognitive abilities of the patients (i.e., insight). On the other hand, it is plausible that more complex sociocognitive functions such as social problem solving are particularly impaired. 

Concerning studies with more than one assessment, to the best of our knowledge only one study assessed influences of an oncological treatment other than surgery. This study reported changes in emotion recognition after radiation [130]. Studies using two time points before and after surgery reported on a performance decrease in emotion recognition in glioblastoma patients [122] as well as region specific impairments of sociocognitive functioning [84] in a heterogeneous group of brain tumor patients. In the latter study, emotion recognition was most strongly impaired in patients with anterior temporal and amygdala lesions. The RMET performance was most strongly impaired in patients with posterior temporoparietal lesions [84]. By contrast, the sensitivity/empathy to others pain was significantly improved in glioma patients postoperatively in another study [127]. Another three studies used three assessments even though these did not extend the second follow-up to more than a few months after surgery [85,97,108]. In low-grade glioma patients a recovery of emotion recognition, cognitive empathy (RMET) [85] and ToM (Comic Strip Task) was observed three [97] and four months after surgery [85] while the patients’ performance declined immediately after surgery [85]. In another study a recovery of emotion recognition abilities was found three months after surgery [108]. Therefore, various authors argued that sociocognitive impairment may be transitory especially in low-grade glioma [85,97,108,110]. However, whether this is related to brain plasticity cannot be inferred from the data presented. Furthermore, additional studies demonstrated an incomplete functional recovery of sociocognitive performance, particularly when the resection cavity was located in specific regions [87,101]. With the aim of functional preservation, a range of studies used intraoperative mapping of sociocognitive functioning [91,110,112,115,119,129]. Based on the findings of these studies, it was argued that intraoperative mapping of social cognition offers added value in brain tumor patients. However, none of those studies assessed complex sociocognitive abilities, such as social problem solving. Therefore, it has not yet been clarified whether intraoperative mapping also preserves higher-order sociocognitive functioning. 

## 4. Discussion

Humans are social beings whose success and satisfaction in daily life relies on cooperation with other social beings to a particular extent [72,73,131]. Supportive social relationships most obviously are especially important for mental health and QoL in brain tumor patients since they suffer from severe health conditions and intensive treatment. For instance, married brain tumor patients were by trend less likely depressed than unmarried individuals [132,133]. This is probably due to overall improved health habits in married individuals with cancer, less delay in seeking medical care when symptomatic and/or greater social support. The ability to maintain supportive social relationships is mediated by sociocognitive functioning (see Section 1). Correspondingly, sociocognitive impairment may result in a variety of interpersonal difficulties such as complaints of frustration in social situations, feelings of social discomfort or feelings of social disconnection and therefore may negatively influence QoL. Due to difficulties in social interactions, visits with friends, family or colleagues may become less frequent during the disease course. As a consequence, patients may experience heightened psychosocial burden and withdraw further from any social function [106]. To this end, the potential consequences of the tumor and/or certain treatments on sociocognitive functioning and psychosocial burden may undermine the “value” of survival [134]. 

### 4.1. Summary of Main Findings

The psychosocial burden in brain tumor patients appears to be moderate to high based on the reviewed primary literature. The Functional Assessment of Cancer Therapy was the most frequently used instrument to assess psychosocial burden although methodology in general was rather heterogenous. Patients may suffer from the influence the brain tumor and its treatment has on their social life and social group membership both immediately after diagnosis [92,93] and also months or years later [83,126]. Role reversals and concerns about the impact of illness on caregivers and losing the ability to care for children were the most frequently reported themes in brain tumor patients [104]. The need for social support was associated with the patients QoL [83] and social support may buffer the effects of multiple treatments and tumor progressions on patients’ wellbeing [102]. 

Contradictory results were yielded by the reviewed studies on sociocognitive functioning in brain tumor patients. Some pretreatment studies reported unimpaired performance or only minor performance deficits in preoperative patients [100,107]. By contrast, other studies pointed to significantly impaired sociocognitive functioning even before any treatment has started in brain tumor patients [88,94]. Likewise, some posttreatment studies reported relevant impairments [87,118,122,130], some region-specific [80,95,101] (e.g., mainly frontal and insular regions). By contrast, other investigations reported no clinically relevant or only minor impairment of sociocognitive functioning in brain tumor patients even after treatment [81,96,98,111,120,127]. 

### 4.2. Clinical and Therapeutical Implications

Although survival as a clear cut and important outcome measure in clinical trials is relevant in Oncology, it does not provide detailed information on the clinical situation of the patient. Since the population of survivors in Neuro-Oncology is growing it is important to gain a more thorough and nuanced understanding of the consequences of brain tumors and their oncological treatment on QoL. Maintaining an acceptable QoL has become a major goal of patient-centered neuro-oncological therapies and constitutes a secondary outcome measure in most clinical oncological trials. 

Brain tumor patients may suffer from overall neurocognitive deficits at some point during the disease course and deficits were detected in about 80% of cases in general [135]. In about 40% of newly diagnosed temporal lobe glioma, deficits in executive functioning were present [136]. In comparison, Goebel et al. (2018) reported a sociocognitive impairment in 83% of patients with at least one of the applied measures [94]. Therefore, in brain tumor patients sociocognitive impairment might be as frequent as general neurocognitive impairment [41,94] but may be rather neglected in research and clinical practice so far. Concerning psychological factors, the reports of depression in patients differed considerably between physicians’ (15%) and brain tumor patients’ (93%) evaluation [137]. This is probably explained by an overestimation of one’s own symptoms or, on the other hand, might be due to the missing of psychological symptoms in clinical settings by simple physician patient interaction. This possibly also applies to psychosocial issues in brain tumor patients since interpersonal difficulties or withdrawal from social interactions are often considered normal reactions in brain tumor patients and are thus not addressed in treatment. As psychosocial burden may interfere with the ability to cope efficiently with cancer, its physical symptoms and its treatment it is important to shed light on these issues in clinical interactions and in future clinical studies. Since sociocognitive dysfunction involves different aspects, which may be impaired independently and also rarely occurs in isolation, different sociocognitive domains should be addressed [138]. Therefore, the “gold-standard” should be a full assessment involving at least one measure for each of the most relevant sociocognitive domains (emotion recognition, empathy, ToM and social problem solving, both in self-/other report and in terms of a performance-based assessment). In particular, there is a need for including ecologically valid measures describing real life situations to capture subtle impairments not detected with some of the laboratory measures [139]. However, since comprehensive neuropsychological assessment is time consuming it may be difficult to include these testing in the routine care of brain tumor patients. Therefore, instruments that appear to be most sensitive to sociocognitive deficits, such as a combination of the RMET and Faux-Pas Task, may be particularly valuable [94]. Furthermore, future studies may implement clinically informed sociocognitive screening questions for physician patient interactions [126] and validate them against comprehensive sociocognitive testing. Those screening questions may enable clinicians to anticipate potential sociocognitive dysfunction, raise the awareness to the need of a comprehensive neuropsychological assessment and guide appropriate diagnostic and treatment. Overall, future studies should validate brief test batteries assessing sociocognitive functions specifically for the use in brain tumor patients (see Ref. [140] for pointing out the relevance of making sure that tests are relevant for a particular clinical group). 

**Figure 2:**
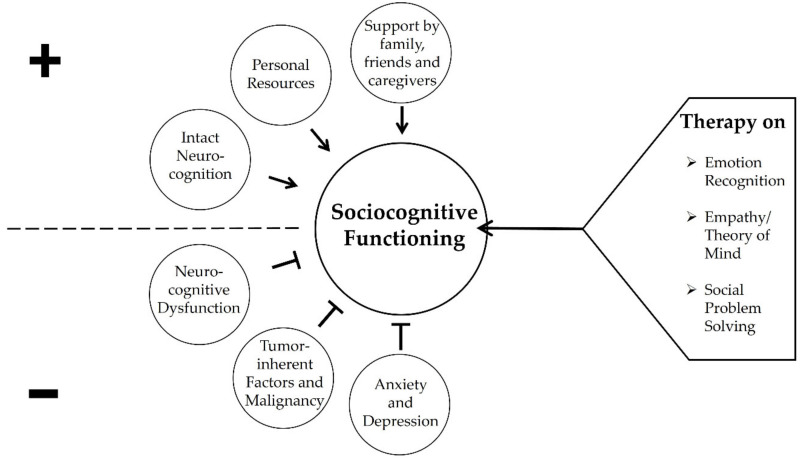
A tentative visualization of parameters that may influence sociocognitive functioning in adult patients with brain tumors and potential targets for therapy. Note. Sociocognitive functioning in brain tumor patients may be negatively affected by the malignancy and location of the tumor (temporal, prefrontal and insular) as well as by the underlying lesion of the central nervous system. By contrast, supportive social relationships with family, friends and other caregivers may positively influence social functioning in brain tumor patients. Since parameters derived from the literature on adult patients with acquired brain injury [59,60,61] may tentatively apply to adult brain tumor patients, intact neurocognition as well as personal resources such as positive problem orientation, coping resources, behavioral and emotional regulation, meta-cognitive abilities and self-awareness may positively influence sociocognitive functioning in adult brain tumor patients. By contrast, the occurrence of mood disorders such as depression and anxiety and neurocognitive dysfunction may negatively affect sociocognitive functioning. These risk and resiliency factors potentially serve as targets for interventions.

In the reviewed literature potential negative effects of malignancy and specific brain tumor locations (i.e., temporal, insular, prefrontal) on sociocognitive abilities were revealed while supportive social relationships and social group membership may positively influence sociocognitive functioning. However, the reviewed primary literature on adult brain tumor patients does not provide clarity on predictive factors for social functioning as yet. Therefore, relevant factors for psychosocial functioning as identified for adults with acquired brain injuries of various etiologies [59,60,61] may also apply to adult brain tumor patients’ sociocognitive functioning. See Figure 2 for a tentative visualization of potential parameters that may affect sociocognitive functioning in adult brain tumor patients as well as potential targets for therapy.

Overall, the available evidence strongly supports the idea of including assessment of social cognition and psychosocial burden into the routine neuropsychological examination in clinical practice and in rehabilitation programs [94,141]. Providing evidence on the prevalence, nature and extent of sociocognitive dysfunction and psychosocial burden may have the potential to inform and direct clinical practice. By allowing clinicians to better anticipate the type of psychosocial problems likely to arise after brain tumor treatment assessment of sociocognitive functioning may lead to more effective supportive (neuropsychotherapeutic) strategies. 

### 4.3. Limitations of Current Research

Overall, to date sociocognitive functioning and psychosocial burden have neither been investigated extensively nor systematically in brain tumor patients and the available data is rather heterogenous preventing a systematic or metanalytic analysis. By the same token, the present review is hampered by the heterogeneity of included studies regarding tumor type, tumor location, type of treatment, time of assessment, methodology applied and the interpretation thereof. Furthermore, there are some shortcomings of the included previous research rendering it difficult to draw firm conclusions from the results. First, the majority of studies reviewed included heterogeneous samples of primary and secondary brain tumors and various entities. Studies with homogeneous samples presented with rather small sample sizes. In the future, larger and/or more homogeneous samples are needed for proper statistical testing and may enable subgroup analyses and/or increase generalizability. Secondly, most of the studies comparing sociocognitive functioning at different time points did not extend testing beyond a few (i.e., three or four) months after surgery [85,97,108] and posttreatment studies had large and variable testing intervals [101]. In studies on psychosocial burden the time intervals are even wider [102,128]. Thirdly, there are some sociocognitive concepts that have been extensively addressed, such as emotion recognition, while social problem solving was targeted only in some studies. Therefore, whether more complex sociocognitive functions such as social problem solving might be impaired while some more basic functions, such as emotion recognition, show only transitory effects of oncological treatment represents an issue meriting further research. In addition, the results of this review may be distorted by the tests administered in the reviewed studies and the fact that laboratory assessments potentially do not adequately reflect the complex sociocognitive demands in everyday social life. However, the tests most frequently applied, such as RMET, Ekman Faces, IRI and Faux-Pas Test provided good psychometric properties in general [138,142]. Nevertheless, some studies included non-standardized measures developed for their own purpose leading to varying psychometric properties [61,142]. Fourthly, there is only a limited number of studies specifically targeting the influence of oncological treatments other than surgery. Although there are posttreatment studies most of them did not assess treatment related factors, with the exception of one study, explicitly gauging the influence of radiation [130]. These aspects require incorporation into future studies to fully understand the impact of each treatment phase and the impact of radio- and chemotherapy on social cognition and psychosocial burden. Fifthly, although the studies assumed an influence of impaired sociocognitive function on QoL, some of these did not explicitly include QoL measures (e.g., Pertz et al., 2021 [118]). Therefore, the influence on QoL should be targeted with specific instruments validated in brain tumor patients, potentially at different time points during the treatment. Furthermore, the assessment of patient reported outcomes (PRO) would have increased the meaningfulness of some parameters influencing sociocognitive functioning. However, PRO have not been comprehensively assessed in the primary literature as summarized in this review. Sixthly, based on the evidence so far it is difficult to separate sociocognitive and neurocognitive disturbances, since they might be interconnected and also share overlapping neural networks [66,143,144]. Since not all the reviewed studies assessed neurocognitive functioning comprehensively, it cannot be completely ruled out that the reported deficits in sociocognitive functioning might be influenced by an underlying deficit in neurocognitive functioning. However, some studies in other clinical groups found that although the demands of some social situations predictably involve cognitive abilities such as planning, monitoring and evaluation, it appears that social-emotional mechanisms may be affected independently [145,146,147]. Finally, to date, it is difficult to differentiate which of the psychosocial symptoms are caused by the neurophysiological effect of the tumor or treatment and which are the patients’ psychological reactions to the stress caused by a serious disease. Consequently, in future research these aspects should be addressed more broadly when addressing sociocognitive functioning and psychosocial burden in cancer patients.

### 4.4. Outlook and Future Directions

The findings of this review suggest that more research efforts are needed to address the specific psychosocial concerns and sociocognitive dysfunction of brain tumor patients. 

Symptoms of impaired social cognition and psychosocial burden may not be revealed in clinical settings by simple physician patient interactions. Similarly as the Mini Mental State Examination is far too brief and insensitive to capture the subtleties of cognitive deficits [148], more comprehensive sociocognitive testing is needed to assess relevant difficulties (see Section 4.2). Therefore, the implementation of easily administered, ecologically valid assessment tools should be introduced in clinical management of brain tumors patients. Sociocognitive assessment should be integrated into larger longitudinal projects to examine its potential to serve as predictor of functional outcomes. 

Studies on other tumor entities (i.e., breast and ovarian cancer) reported that the support of family and friends is vital. People who were socially isolated when diagnosed with cancer are more likely to die than those with strong social networks. This relationship was attributed to a lack of access to care, beneficial caregiving from friends or relatives as well as to the consideration of support activities during adjuvant treatment [149,150]. Especially for adults of working age, with a partner, children, family and financial responsibilities, a cancer diagnosis adversely impacts on the person’s life. Therefore, an important aspect of future research may also be the assessment of sociocognitive functions in patients with non-central nervous system cancer, especially in those of younger age. 

Furthermore, brain tumor patients may benefit from having a single point of contact during the disease. However, in current daily practice neuro-oncological treatments are applied by different disciplines and specialists in sequence. Since the psychosocial support of patients comes up short the installation of a neuro-oncologist as a “patient-guide” represents a feasible opportunity to support the patients and their caregivers during the disease course. In the future, interdisciplinary collaborations have the potential not only to address the physical and cognitive issues of brain tumor patients but also to focus on emotional and social needs.

Furthermore, it is not only the patients who might show significant burden due to sociocognitive impairments but also the patients’ caregivers might suffer from decreases in QoL. A brain tumor diagnosis was explained as a “family disease” that has been found to result in major changes to relationships and high level of caregiver strain [151,152,153]. The support persons may be burdened by changes in relationships or family dynamics and fear of losing their loved one [154,155,156]. Furthermore, patients’ impairment in sociocognitive functioning and an increased psychosocial burden may hinder their social functioning, potentially contributing to social isolation of both the patients and their caregivers. 

Given a link between sociocognitive impairment and well-being treatment of the respective dysfunction may be of interest. However, literature on sociocognitive treatment in the brain tumor population is extremely rare. By contrast, a range of studies exist evaluating sociocognitive treatments for psychiatric disorders such as schizophrenia, bipolar and autism spectrum disorders [157]. A recent review on patients with traumatic brain injury recommend that treatment should include comprehensive programs addressing the most relevant aspects of social cognition (i.e., emotion recognition, empathy/ToM and social communication), tailored to each patients’ specific deficits [158]. In addition, direct interactive treatments, e.g., based on role play techniques, particularly with group therapy settings, may be beneficial [159]. On the other hand, incorporation of technologically supported therapeutic elements (e.g., virtual reality based, internet- and app-based programs), already in use in various psychiatric and neurological populations, might represent an asset in the treatment of sociocognitive dysfunction in brain tumor patients suffering from an increased psychosocial burden (see Ref. [140] for cerebrovascular diseases). These therapeutic elements provide the opportunity to practice real life-type social scenarios without the pressure that actual social interactions in real-time entail and still facilitate generalization to real-word interactions in later stages. Future research may assess whether these treatments of sociocognitive dysfunction validated in psychiatric populations [160,161] or in patients after traumatic brain injuries [162] may be appropriate for brain tumor patients. Since previous studies demonstrated the helpful influence of psychotherapeutic interventions [102,114] and one study assessed the impact of a non-specific problem-solving cancer care education for patients and caregivers [163], future research should address the applicability of these interventions in brain tumor patients. In addition, certain risk factors for psychosocial burden or especially vulnerable groups of patients [79,103,121,128] were identified. Recently, a study indicated that in brain tumor patients who were classified as distressed a psychological intervention improved well-being while in patients not classified as distressed no changes were noted [164]. Therefore, treatment programs targeting psychosocial burden and sociocognitive dysfunction need to carefully distinguish between different components of the relevant concepts and need to take into account the specific characteristics and patterns of impairments of this particular patient group. 

## 5. Conclusions

Brain tumor survivorship has become an increasingly important area of neuro-oncological care since survival rates are increasing and these patients represent a vulnerable population with distinct medical, psychosocial, emotional and (socio)cognitive needs. These needs may potentially change during the course of the disease, negatively impact QoL and add an additional burden to caregivers and the patients themselves. Although in the reviewed studies the evidence of the impact of brain tumors and their treatment on sociocognitive functions and psychosocial burden is inconsistent, the majority of the studies suggest an increased psychosocial burden in the patients. Furthermore, some studies indicated that sociocognitive functions might be as frequently impaired as classical neurocognitive functions. Therefore, certain aspects of needs of brain tumor patients and their social environment may be unmet until now: social cognition and psychosocial burden potentially represent an overseen area in oncological research so far. Since sociocognitive deficits represent a potentially modifiable factor [165], social cognition should be assessed more broadly. There is the need to raise awareness for these sociocognitive difficulties among clinicians, researchers and patients alongside the more established aspects of neurocognition in order to improve patients’ and caregivers’ QoL.

## Figures and Tables

**Table 1:** Summary of the topic, design and main instruments to assess sociocognitive functioning and psychosocial burden of the identified studies.

Authors	Topic	Design	Main Instrument of Sociocognitive Functioning or Psychosocial Burden
Andrewes et al. (2003) [79]	psychosocial burden	crosssectional	emotional and social dysfunction questionnaire
Baird et al. (2006) [80]	social cognition	crosssectional	facial emotional expression multimorph task: recognition of a neutral face gradually morphed through twenty 5% increment stages into 1 of 6 prototypical expressions (happiness, sadness, anger, disgust, fear and surprise); social situations task: judge the appropriateness of behaviors in short stories of social situations (normative versus violation); joke interpretation: state whether the scenario was amusing and why (correct answers referred directly to the thoughts, feelings and dispositions of the characters); advanced ToM: interpret and justify the behavior of the main protagonist in stories of naturalistic social situations
Baird et al. (2014) [81]	social cognition	case study	emotions portrayed by music excerpts (happy, peaceful, sad and scary); the Awareness of Social Inferences Test (happy, surprised, neutral, sad, angry, anxious)
Bowers & Heilman (1984) [82]	social cognition	case study	Neutral Facial Discrimination Task: state whether 2 faces (unfamiliar with neutral facial expression) were the same or a different person; Name the Facial Emotion Task: name the facial emotion depicted in a photograph with 1 of 4 facial emotions (happiness, sadness, anger or indifference); Choose the Facial Emotion Task: point to the face that depicted a target emotion named by the examiner (i.e., point to the sad face); Same-Different Facial Emotion Task: indicated whether the emotion portrayed by 2 same faces was the same or different; Affective Prosody Task: identify the affective intonation of a sentence (semantically neutral sentences recorded in 4 different affective intonations: happy, sad, angry, indifferent)
Bunston et al. (1998) [83]	psychosocial burden	crosssectional	Coping in stressful Situations Scale to measure 3 major coping styles: task-oriented, emotion-oriented and avoidance coping; FACT-Brain (subscales: physical, functional, social/family, emotional well-being, relationship with doctor, total score); Fatigue Severity Scale; The Life Event Survey to measure life event stress by assessing both the extent of desirability and personal impact; The Princess Margaret Hospital Needs Assessment Inventory to identify 58 specific needs grouped into 12 domains of need
Campanella et al. (2014) [84]	social cognition	prospective	Emotion recognition (Ekman Faces) word-to-picture matching task: 6 faces of the same person expressing 6 basic emotions (happiness, sadness, anger, surprise, fear and disgust); RMET; Toronto Alexithymia Scale; Temperament and Character Inventory
Campanella et al. (2015) [85]	social cognition	prospective	Emotion recognition (Ekman Faces) word-to-picture matching task: 6 faces of the same person expressing 6 basic emotions (happiness, sadness, anger, surprise, fear and disgust); RMET; Toronto Alexithymia Scale; Temperament and Character Inventory
Cavers et al. (2012) [86]	psychosocial burden	prospective	qualitative longitudinal multiperspective technique; interviews conducted over a period of 2 years to explore the experiences of patients and caregivers
Channon et al. (2007) [87]	social cognition	crosssectional	pragmatic comprehension task: social context with 4 different types of endings (control physical event, human action, direct sarcastic remark, indirect sarcastic remark), generation of appropriate interpretations of the final remark; selection of best interpretation among alternatives
Chen et al. (2016) [88]	social cognition	crosssectional	IRI; forced-choice facial Emotion Recognition Task with 5 basic emotions and neutral; perception of others’ pain task; emotional perspective taking; Toronto Alexithymia Scale
Cornwell et al. (2012) [89]	psychosocial burden	prospective	semi-structured interview with open questions; perspectives on issues related to patients’ health; in-depth interview asked about experiences and feelings of life at home since discharge, ongoing therapy and support services, perceived needs and barriers and facilitators to goal achievement
Cubis et al. (2019) [90]	psychosocial burden	crosssectional	FACT-cognitive function and general; The Exeter Identity Transition Scale (pre-existing social groups, the maintenance of social groups and new social groups); Social Subscale from the Traumatic Brain Injury Self-Efficacy Scale (confidence in support from social group membership); Satisfaction with Life Scale; the seven item depression Scale of Depression Anxiety Stress Scales; The Generalized Anxiety Disorder Scale
Giussani et al. (2010) [91]	social cognition	prospective	identify and name facial emotion expression (anger, happiness, fear, surprise, disgust and sadness); intraoperative facial emotion recognition task (anger, happiness, fear, surprise, disgust and sadness)
Goebel et al. (2011) [92]	psychosocial burden	crosssectional	clinical interview for diagnostic and statistical manual of mental disorders fourth edition; distress thermometer; HADS; Impact of Event Scale-revised; questionnaire to mark distressing events during illness
Goebel et al. (2011) [93]	psychosocial burden	crosssectional	distress thermometer and associated problem list of the distress thermometer (practical, family, emotional, spiritual-religious or physical problems); HADS, Questionnaire for the Assessment of social support
Goebel et al. (2018) [94]	social cognition and psychosocial burden	crosssectional	Karolinska directed emotional faces (emotion recognition, facial differentiation, emotional differentiation); ToM with the RMET; complex ToM reasoning with the Faux-Pas Test; nonverbal cognitive and affective ToM with Picture Stories; Empathy quotient; HADS; Marburg Competence Scale; Social Adjustment Scale; Social and occupational functional assessment scale (examiners rating)
Gu et al. (2012) [95]	social cognition	crosssectional	empathy for pain paradigm with explicit pain condition: judge whether the person in the photograph was suffering from pain or not and implicit pain condition: judge the laterality of the hand/foot
Guha-Thakurta et al. (1999) [96]	social cognition	crosssectional	modified FACT-Brain; Symptom Questionnaire; Social Adjustment Scale Self-Report; Problem Solving Inventory
Herbet et al. (2013) [97]	social cognition	prospective	RMET; Comic Strip Task
Herbet et al. (2014) [98]	social cognition	crosssectional	RMET; Comic Strip Task
Herbet et al. (2015) [99]	social cognition	crosssectional	Empathy quotient
Herbet et al. (2015) [100]	social cognition	crosssectional	RMET (preoperative: 4 response options, intraoperative: 2 response options)
Jenkins et al. (2014) [101]	social cognition	crosssectional	Emotion recognition Task: facial morphing with neutral faces changing into emotional (anger, disgust, fear, happiness, sadness and surprise) faces of differing intensities (20–100%); Perspective Taking Task: ToM scale (inferences on thoughts of the character), empathy scale (inferences on feelings of the character), physical scale (inferences on physical events)
Kangas et al. (2011) [102]	psychosocial burden	crosssectional	The Profile of Mood States; The Intrusion and Avoidance Subscale from the Impact of Event Scale-Revised; The Multidimensional Scale of Perceived Social Support
Kangas et al. (2012) [103]	psychosocial burden	prospective	Post-Traumatic Stress Disorder Checklist-Stressor Specific Version (group categorization in high and low symptoms); Impact of Event Scale-Revised; FACT-General and Brain; Profile of Mood States; Partner Response to Cancer Inventory (perceived positive support); Social Constraints Scale
Kanter et al. (2014) [104]	psychosocial burden	crosssectional	quantitative analyses of themes discussed in support groups
Langbecker & Yates (2016) [105]	psychosocial burden	prospective	Katz Index of Independence in Activities of daily living; Lwanton-Brody Instrumental Activities of daily living; Supportive Care Needs Survey short form and brain tumor-specific items; Distress thermometer; FACT-Brain
Lucas (2010) [106]	psychosocial burden	qualitative study	hundreds of unstructured interviews conducted between 2001–2008 in individual settings and in the group context
Luherne-du Boullay et al. (2014) [107]	social cognition	crosssectional	visual emotional recognition task from the Karolinska Directed Emotional Faces (happiness, sadness, disgust, anger, fear and neutral face); auditory emotional recognition task with 60 affect vocalizations (happiness, sadness, disgust, anger, fear and neutral) from the Montréal Affective Voices; crossmodal stimuli: emotional faces and voices congruently and simultaneously presented
Mattavelli et al. (2017) [108]	social cognition	prospective	Ekman 60 Faces test: recognition of emotional facial expressions (matching to sample procedure; surprise, happiness, fear, disgust, anger and sadness); recognition of emotion from prosody: new experimental paradigm (sentences consisting of pseudo-words with a prosody corresponding to 1 of 6 emotions)
Mu et al. (2012) [109]	social cognition	case-control study	Facial Expression Identification: photos from the Chinese static facial expression gallery with 6 types of basic emotions and neutral expressions
Nakajima et al. (2018) [110]	social cognition	prospective	intraoperative mentalizing test: False Belief Task; Cartoon format of the picture arrangement Task of the WAIS third edition
Nakajima et al. (2018) [111]	social cognition	crosssectional	RMET
Nakajima et al. (2021) [112]	social cognition	prospective	Intraoperative Basic Emotional Test with photos of modified Japanese facial expression of basic emotional series (eye region): selection of most suitable emotional state from 2 choices within 2 seconds; Expression Recognition Test for adults with 32 photographs of basic emotions (happiness, sadness, anger and surprise): selection of most reasonable mental state from 5 choices
Ownsworth et al. (2011) [113]	psychosocial burden	crosssectional	in depth semi-structured interviews
Ownsworth et al. (2015) [114]	psychosocial burden	randomized wait-list control study	McGill QoL Questionnaire: physical, psychological, existential and social well-being; Montgomery-Asberg Depression Rating Scale; Depression Anxiety Stress Scales-21; FACT-Brain
Papagno et al. (2016) [115]	social cognition	prospective	Forced-choice Emotion recognition Task (stimuli selected from FEEST to create a modified version of the Ekman test): selection of correct emotion among 5 alternatives written below the picture (orally or pointing), emotions of anger, fear, happiness, disgust (excluding sadness and surprise) and a mildly neutral expression (happiness at 25% of its intensity)
Peper & Irle (1997) [116]	social cognition	crosssectional	selection of category labels: name and select the correct label on a multiple-choice card of presented emotional vocalizations joy, anxiety, sadness and anger (unimodal multiple choice-task); crossmodal vocal-visual recognition of emotion categories with matching emotion categories between auditory and visual stimuli (matching to sample procedure): vocal probe stimulus followed by 2 Ekman & Friesen photographs displaying the same emotion category or a new category to choose from; recognition of affiliative emotion dimensions (valence and arousal) with matching emotion dimensions between auditory and visual stimuli: vocal probe stimulus displaying one emotion category followed by 2 photographs with 2 different emotion categories with corresponding emotion dimensions or not
Peper & Irle (1997) [117]	social cognition	crosssectional	selection of category labels: name and select the correct category label on a multiple-choice card of pictures (Ekman and Friesen’s Pictures of Facial Affect) displaying emotional categories (happiness, surprise, anger, anxiety, grief and disgust); selection of named emotion category: select the facial expression (6 emotional expressions of different faces) named by the examiner; matching emotion categories: matching to sample paradigm with 6 categories and an additional neutral stimulus (presentation of probe stimulus immediately followed by 2 choice photographs with the same emotion category and a new category to choose from); matching emotion dimension: probe face displaying one emotion category and 2 response photographs displaying 2 different emotion categories, with either a corresponding emotion dimension or not
Pertz et al. (2021) [118]	social cognition	crosssectional	IRI; Multifaceted Empathy Test; Social Problem Solving Fluency Task: ability to detect and interpret awkwardness in hypothetical real-life social situations; discomfort experienced in problematic social situations; capacity to freely generate and merely recognize appropriate solutions for social problems
Prat-Acin et al. (2021) [119]	social cognition	prospective	modified version of RMET
Saver & Damasio (1991) [120]	social cognition	case study	The Optional Thinking Test (ability to generate alternative solutions to hypothetical social dilemmas); The Awareness of Consequences Test (spontaneous inclination to consider the consequences of social actions); The Means-End Problem-solving procedure (ability to conceptualize efficacious step-by-step means to achieve social goals); The Carton Prediction Test (ability to predict the social consequences of events)
Shin et al. (2016) [121]	psychosocial burden	crosssectional	qualitative interview with semi-structured questions; questions included “How have your seizures affected your relationships?”
Sinha et al. (2020) [122]	social cognition	prospective	Affective Facial Expression Test: selection of correct emotional expression in faces (happiness, sadness, anger, surprise, fear, disgust); patient health questionnaire
Szelag & Fersten (1991) [123]	social cognition	crosssectional	emotion recognition with faces expressing positive, negative (happy and sad) and neutral emotions in a visual half field paradigm (left or right from a fixation point), effectiveness of perception in the left and right visual fields measured by number of errors
Trejnowska et al. (2020) [124]	psychosocial burden	crosssectional	Mini-Mental Adjustment to Cancer Scale; Experiences in Close Relationships-Revised questionnaire; Modified Medical Outcomes Study-Social Support Scale; FACT-Brain-physical well-being
Troschel et al. (2021) [125]	psychosocial burden	prospective	personal behavior (i.e., number of weekly contacts to friends, acquaintances, or family outside the home environment independent of contact in person, via telephone or via video tools); Isolation Questionnaire; HADS; Distress Thermometer; WHO5 well-being score
Voß et al. (2021) [126]	psychosocial burden	crosssectional	patient interviews covering 6 main areas: psyche, cognition, body, role functioning, social support, unmet needs; rating whether the issues affected them and the importance of these areas
Wang et al. (2014) [127]	social cognition	prospective	Empathy For Others Pain Task with pain condition and laterality condition; IRI; Toronto Alexithymia Scale
Weitzner et al. (1996) [128]	psychosocial burden	crosssectional	Ferrans and Powers QoL Index for Cancer (health and functioning, socioeconomic aspects, psychological/spiritual aspects, family); Psychosocial Adjustment to Illness Scale-Self Report (healthcare orientation, vocational environment, domestic environment, sexual relationships, extended family relationships, social environment, psychological distress)
Yordanova et al. (2017) [129]	social cognition	prospective	RMET; modified version of the RMET (only 2 mental state options, for each patient items with a wrong answer during preoperative assessment were excluded)
Yuksek et al. (2015) [130]	social cognition	prospective	Facial Emotion Recognition Test with Ekman and Friesen’s Faces (happy, surprised, fearful, sad, angry, disgusted and neutral facial expression)

Note. Facial Expressions of Emotion: Stimuli and Tests (FEEST), Hospital Anxiety and Depression Scale (HADS), Interpersonal Reactivity Index (IRI), Quality of Life (QoL), Reading the Mind in the Eyes Test (RMET), The Functional Assessment of Cancer Therapy (FACT), Theory of Mind (ToM), Wechsler Adult Intelligence Scale Revised (WAIS), World Health Organization (WHO).

## Data Availability

Not applicable.

## Supplementary Materials

The following supporting information can be downloaded at: https://www.mdpi.com/article/10.3390/cancers14030767/s1, Table S1: Summary of the time of assessment, number of patients, control group and matching criteria as well as key findings concerning sociocognitive functioning or psychosocial burden of the identified studies.

## References

[1] Lieberman A.N., Foo S.H., Ransohoff J., Wise A., George A., Gordon W., Walker R. (1982). Long term survival among patients with malignant brain tumors. Neurosurgery.

[2] Boele F.W., Douw L., Reijneveld J.C., Robben R., Klein M. (2015). Health-Related Quality of Life in Stable, Long-Term Survivors of Low-Grade Glioma. J. Clin. Oncol..

[3] Beier D., Proescholdt M., Reinert C., Pietsch T., Jones D.T.W., Pfister S.M., Hattingen E., Seidel C., Dirven L., Luerding R. (2018). Multicenter pilot study of radiochemotherapy as first-line treatment for adults with medulloblastoma (NOA-07). Neuro-Oncology.

[4] Seidel S., Pels H., Schlömer S., Kowoll A., Fliessbach K., Engert A., Vogt-Schaden M., Egerer G., Reichmann H., Schackert G. (2020). Twenty-year follow-up of a pilot/phase II trial on the Bonn protocol for primary CNS lymphoma. Neurology.

[5] Herrlinger U., Tzaridis T., Mack F., Steinbach J.P., Schlegel U., Sabel M., Hau P., Kortmann R.-D., Krex D., Grauer O. (2019). Lomustine-temozolomide combination therapy versus standard temozolomide therapy in patients with newly diagnosed glioblastoma with methylated MGMT promoter (CeTeG/NOA–09): A randomised, open-label, phase 3 trial. Lancet.

[6] Olson J.D., Riedel E., DeAngelis L.M. (2000). Long-term outcome of low-grade oligodendroglioma and mixed glioma. Neurology.

[7] Weller J., Tzaridis T., Mack F., Steinbach J.P., Schlegel U., Hau P., Krex D., Grauer O., Goldbrunner R., Bähr O. (2019). Health-related quality of life and neurocognitive functioning with lomustine–temozolomide versus temozolomide in patients with newly diagnosed, MGMT-methylated glioblastoma (CeTeG/NOA-09): A randomised, multicentre, open-label, phase 3 trial. Lancet Oncol..

[8] Klein M. (2012). Neurocognitive functioning in adult WHO grade II gliomas: Impact of old and new treatment modalities. Neuro-Oncology.

[9] Taphoorn M.J.B., Klein M. (2004). Cognitive deficits in adult patients with brain tumours. Lancet Neurol..

[10] Fountain D.M., Allen D., Joannides A.J., Nandi D., Santarius T., Chari A. (2016). Reporting of patient-reported health-related quality of life in adults with diffuse low-grade glioma: A systematic review. Neuro-Oncology.

[11] Butenschoen V.M., Kelm A., Meyer B., Krieg S.M. (2019). Quality-adjusted life years in glioma patients: A systematic review on currently available data and the lack of evidence-based utilities. J. Neurooncol..

[12] Coomans M.B., Dirven L., Aaronson N.K., Baumert B.G., van den Bent M., Bottomley A., Brandes A.A., Chinot O., Coens C., Gorlia T. (2020). Calculating the net clinical benefit in neuro-oncology clinical trials using two methods: Quality-adjusted survival effect sizes and joint modeling. Neurooncol. Adv..

[13] Buckner J.C., Shaw E.G., Pugh S.L., Chakravarti A., Gilbert M.R., Barger G.R., Coons S., Ricci P., Bullard D., Brown P.D. (2016). Radiation plus Procarbazine, CCNU, and Vincristine in Low-Grade Glioma. N. Engl. J. Med..

[14] Juergens A., Pels H., Rogowski S., Fliessbach K., Glasmacher A., Engert A., Reiser M., Diehl V., Vogt-Schaden M., Egerer G. (2010). Long-term survival with favorable cognitive outcome after chemotherapy in primary central nervous system lymphoma. Ann. Neurol..

[15] Lawrie T.A., Gillespie D., Dowswell T., Evans J., Erridge S., Vale L., Kernohan A., Grant R. (2019). Long-term neurocognitive and other side effects of radiotherapy, with or without chemotherapy, for glioma. Cochrane Database Syst. Rev..

[16] Brown P.D., Buckner J.C., O’Fallon J.R., Iturria N.L., Brown C.A., O’Neill B.P., Scheithauer B.W., Dinapoli R.P., Arusell R.M., Curran W.J. (2003). Effects of radiotherapy on cognitive function in patients with low-grade glioma measured by the folstein mini-mental state examination. J. Clin. Oncol..

[17] Jalali R., Gupta T., Goda J.S., Goswami S., Shah N., Dutta D., Krishna U., Deodhar J., Menon P., Kannan S. (2017). Efficacy of Stereotactic Conformal Radiotherapy vs Conventional Radiotherapy on Benign and Low-Grade Brain Tumors: A Randomized Clinical Trial. JAMA Oncol..

[18] Kiebert G.M., Curran D., Aaronson N.K., Bolla M., Menten J., Rutten E.H., Nordman E., Silvestre M.E., Pierart M., Karim A.B. (1998). Quality of life after radiation therapy of cerebral low-grade gliomas of the adult: Results of a randomised phase III trial on dose response (EORTC trial 22844). EORTC Radiotherapy Co-operative Group. Eur. J. Cancer.

[19] Klein M., Heimans J.J., Aaronson N.K., van der Ploeg H.M., Grit J., Muller M., Postma T.J., Mooij J.J., Boerman R.H., Beute G.N. (2002). Effect of radiotherapy and other treatment-related factors on mid-term to long-term cognitive sequelae in low-grade gliomas: A comparative study. Lancet.

[20] Douw L., Klein M., Fagel S.S., van den Heuvel J., Taphoorn M.J.B., Aaronson N.K., Postma T.J., Vandertop W.P., Mooij J.J., Boerman R.H. (2009). Cognitive and radiological effects of radiotherapy in patients with low-grade glioma: Long-term follow-up. Lancet Neurol..

[21] Prabhu R.S., Won M., Shaw E.G., Hu C., Brachman D.G., Buckner J.C., Stelzer K.J., Barger G.R., Brown P.D., Gilbert M.R. (2014). Effect of the addition of chemotherapy to radiotherapy on cognitive function in patients with low-grade glioma: Secondary analysis of RTOG 98-02. J. Clin. Oncol..

[22] Reijneveld J.C., Taphoorn M.J.B., Coens C., Bromberg J.E.C., Mason W.P., Hoang-Xuan K., Ryan G., Hassel M.B., Enting R.H., Brandes A.A. (2016). Health-related quality of life in patients with high-risk low-grade glioma (EORTC 22033-26033): A randomised, open-label, phase 3 intergroup study. Lancet Oncol..

[23] Vigliani M.-C., Sichez N., Poisson M., Delattre J.-Y. (1996). A prospective study of cognitive functions following conventional radiotherapy for supratentorial gliomas in young adults: 4-year results. Int. J. Radiat. Oncol. Biol. Phys..

[24] Habets E.J.J., Taphoorn M.J.B., Nederend S., Klein M., Delgadillo D., Hoang-Xuan K., Bottomley A., Allgeier A., Seute T., Gijtenbeek A.M.M. (2014). Health-related quality of life and cognitive functioning in long-term anaplastic oligodendroglioma and oligoastrocytoma survivors. J. Neurooncol..

[25] Taphoorn M.J.B., van den Bent M.J., Mauer M.E.L., Coens C., Delattre J.-Y., Brandes A.A., Sillevis Smitt P.A.E., Bernsen H.J.J.A., Frénay M., Tijssen C.C. (2007). Health-related quality of life in patients treated for anaplastic oligodendroglioma with adjuvant chemotherapy: Results of a European Organisation for Research and Treatment of Cancer randomized clinical trial. J. Clin. Oncol..

[26] Wang M., Cairncross G., Shaw E., Jenkins R., Scheithauer B., Brachman D., Buckner J., Fink K., Souhami L., Laperriere N. (2010). Cognition and quality of life after chemotherapy plus radiotherapy (RT) vs. RT for pure and mixed anaplastic oligodendrogliomas: Radiation therapy oncology group trial 9402. Int. J. Radiat. Oncol. Biol. Phys..

[27] Correa D.D., Shi W., Thaler H.T., Cheung A.M., DeAngelis L.M., Abrey L.E. (2008). Longitudinal cognitive follow-up in low grade gliomas. J. Neurooncol..

[28] Surma-aho O., Niemelä M., Vilkki J., Kouri M., Brander A., Salonen O., Paetau A., Kallio M., Pyykkönen J., Jääskeläinen J. (2001). Adverse long-term effects of brain radiotherapy in adult low-grade glioma patients. Neurology.

[29] Gondi V., Hermann B.P., Mehta M.P., Tomé W.A. (2012). Hippocampal dosimetry predicts neurocognitive function impairment after fractionated stereotactic radiotherapy for benign or low-grade adult brain tumors. Int. J. Radiat. Oncol. Biol. Phys..

[30] Wefel J.S., Cloughesy T., Zazzali J.L., Zheng M., Prados M., Wen P.Y., Mikkelsen T., Schiff D., Abrey L.E., Yung W.K.A. (2011). Neurocognitive function in patients with recurrent glioblastoma treated with bevacizumab. Neuro-Oncology.

[31] Armstrong T.S., Wefel J.S., Wang M., Gilbert M.R., Won M., Bottomley A., Mendoza T.R., Coens C., Werner-Wasik M., Brachman D.G. (2013). Net clinical benefit analysis of radiation therapy oncology group 0525: A phase III trial comparing conventional adjuvant temozolomide with dose-intensive temozolomide in patients with newly diagnosed glioblastoma. J. Clin. Oncol..

[32] Wefel J.S., Kayl A.E., Meyers C.A. (2004). Neuropsychological dysfunction associated with cancer and cancer therapies: A conceptual review of an emerging target. Br. J. Cancer.

[33] Correa D.D., DeAngelis L.M., Shi W., Thaler H., Glass A., Abrey L.E. (2004). Cognitive functions in survivors of primary central nervous system lymphoma. Neurology.

[34] Fliessbach K., Helmstaedter C., Urbach H., Althaus A., Pels H., Linnebank M., Juergens A., Glasmacher A., Schmidt-Wolf I.G., Klockgether T. (2005). Neuropsychological outcome after chemotherapy for primary CNS lymphoma: A prospective study. Neurology.

[35] Fliessbach K., Urbach H., Helmstaedter C., Pels H., Glasmacher A., Kraus J.A., Klockgether T., Schmidt-Wolf I., Schlegel U. (2003). Cognitive performance and magnetic resonance imaging findings after high-dose systemic and intraventricular chemotherapy for primary central nervous system lymphoma. Arch. Neurol..

[36] Abrey L.E. (2012). The impact of chemotherapy on cognitive outcomes in adults with primary brain tumors. J. Neurooncol..

[37] Froklage F.E., Oosterbaan L.J., Sizoo E.M., de Groot M., Bosma I., Sanchez E., Douw L., Heimans J.J., Reijneveld J.C., Lagerwaard F.J. (2014). Central neurotoxicity of standard treatment in patients with newly-diagnosed high-grade glioma: A prospective longitudinal study. J. Neurooncol..

[38] Torres I.J., Mundt A.J., Sweeney P.J., Llanes-Macy S., Dunaway L., Castillo M., Macdonald R.L. (2003). A longitudinal neuropsychological study of partial brain radiation in adults with brain tumors. Neurology.

[39] Laack N.N., Brown P.D., Ivnik R.J., Furth A.F., Ballman K.V., Hammack J.E., Arusell R.M., Shaw E.G., Buckner J.C. (2005). Cognitive function after radiotherapy for supratentorial low-grade glioma: A North Central Cancer Treatment Group prospective study. Int. J. Radiat. Oncol. Biol. Phys..

[40] Talacchi A., Santini B., Savazzi S., Gerosa M. (2011). Cognitive effects of tumour and surgical treatment in glioma patients. J. Neurooncol..

[41] Tucha O., Smely C., Preier M., Lange K.W. (2000). Cognitive deficits before treatment among patients with brain tumors. Neurosurgery.

[42] Lageman S.K., Cerhan J.H., Locke D.E.C., Anderson S.K., Wu W., Brown P.D. (2010). Comparing neuropsychological tasks to optimize brief cognitive batteries for brain tumor clinical trials. J. Neurooncol..

[43] Hahn C.A., Dunn R.H., Logue P.E., King J.H., Edwards C.L., Halperin E.C. (2003). Prospective study of neuropsychologic testing and quality-of-life assessment of adults with primary malignant brain tumors. Int. J. Radiat. Oncol. Biol. Phys..

[44] Bosma I., Douw L., Bartolomei F., Heimans J.J., van Dijk B.W., Postma T.J., Stam C.J., Reijneveld J.C., Klein M. (2008). Synchronized brain activity and neurocognitive function in patients with low-grade glioma: A magnetoencephalography study. Neuro-Oncology.

[45] Ek L., Almkvist O., Wiberg M.K., Stragliotto G., Smits A. (2010). Early cognitive impairment in a subset of patients with presumed low-grade glioma. Neurocase.

[46] Krupp W., Klein C., Koschny R., Holland H., Seifert V., Meixensberger J. (2009). Assessment of neuropsychological parameters and quality of life to evaluate outcome in patients with surgically treated supratentorial meningiomas. Neurosurgery.

[47] Ownsworth T. (2016). Coping with the Unthinkable: Psychosocial Advances in the Management of Primary Brain Tumour. Brain Impair..

[48] Loughan A.R., Lanoye A., Aslanzadeh F.J., Villanueva A.A.L., Boutte R., Husain M., Braun S. (2021). Fear of Cancer Recurrence and Death Anxiety: Unaddressed Concerns for Adult Neuro-oncology Patients. J. Clin. Psychol. Med. Settings.

[49] Carlson L.E., Angen M., Cullum J., Goodey E., Koopmans J., Lamont L., MacRae J.H., Martin M., Pelletier G., Robinson J. (2004). High levels of untreated distress and fatigue in cancer patients. Br. J. Cancer.

[50] Keir S.T., Calhoun-Eagan R.D., Swartz J.J., Saleh O.A., Friedman H.S. (2008). Screening for distress in patients with brain cancer using the NCCN’s rapid screening measure. Psychooncology.

[51] Riba M.B., Donovan K.A., Andersen B., Braun I., Breitbart W.S., Brewer B.W., Buchmann L.O., Clark M.M., Collins M., Corbett C. (2019). Distress Management, Version 3.2019, NCCN Clinical Practice Guidelines in Oncology. J. Natl. Compr. Cancer Netw..

[52] Randazzo D.M., McSherry F., Herndon J.E., Affronti M.L., Lipp E.S., Flahiff C., Miller E., Woodring S., Freeman M., Healy P. (2017). A cross sectional analysis from a single institution’s experience of psychosocial distress and health-related quality of life in the primary brain tumor population. J. Neurooncol..

[53] Ownsworth T., Henderson L., Chambers S.K. (2010). Social support buffers the impact of functional impairments on caregiver psychological well-being in the context of brain tumor and other cancers. Psychooncology.

[54] Ozbay F., Johnson D.C., Dimoulas E., Morgan C.A., Charney D., Southwick S. (2007). Social Support and Resilience to Stress: From Neurobiology to Clinical Practice. Psychiatry.

[55] Payne S., Jarrett N., Jeffs D., Brown L. (2001). Implications of social isolation during cancer treatment. The implications of residence away from home during cancer treatment on patients’ experiences: A comparative study. Health Place.

[56] Cobb S. (1976). Social support as a moderator of life stress. Psychosom. Med..

[57] House J.S., Landis K.R., Umberson D. (1988). Social relationships and health. Science.

[58] Berkman L.F., Syme S.L. (1979). Social networks, host resistance, and mortality: A nine-year follow-up study of Alameda County residents. Am. J. Epidemiol..

[59] Mcdonald S., Genova H. (2021). The Effect of Severe Traumatic Brain Injury on Social Cognition, Emotion Regulation, and Mood.

[60] Kendall E. (1996). Psychosocial Adjustment Following Closed Head Injury: A Model for Understanding Individual Differences and Predicting Outcome. Neuropsychol. Rehabil..

[61] Cassel A., Mcdonald S., Kelly M., Togher L. (2016). Learning from the minds of others: A review of social cognition treatments and their relevance to traumatic brain injury. Neuropsychol. Rehabil..

[62] Keyes C.L.M. (1998). Social Well-Being. Soc. Psychol. Q..

[63] Adolphs R. (2001). The neurobiology of social cognition. Curr. Opin. Neurobiol..

[64] Frith C.D. (2008). Social cognition. Philos. Trans. R. Soc. Lond. Ser. B Biol. Sci..

[65] Adolphs R. (2002). Neural systems for recognizing emotion. Curr. Opin. Neurobiol..

[66] Decety J., Lamm C. (2006). Human empathy through the lens of social neuroscience. Sci. World J..

[67] Shamay-Tsoory S.G., Aharon-Peretz J., Perry D. (2009). Two systems for empathy: A double dissociation between emotional and cognitive empathy in inferior frontal gyrus versus ventromedial prefrontal lesions. Brain.

[68] Dvash J., Shamay-Tsoory S.G. (2014). Theory of Mind and Empathy as Multidimensional Constructs. Top. Lang. Disord..

[69] Shamay-Tsoory S.G., Aharon-Peretz J. (2007). Dissociable prefrontal networks for cognitive and affective theory of mind: A lesion study. Neuropsychologia.

[70] Chang E.C., D’Zurilla T.J., Sanna L.J. (2004). Social Problem Solving: Theory, Research, and Training.

[71] D’Zurilla T.J., Nezu A.M. (1990). Development and preliminary evaluation of the Social Problem-Solving Inventory. Psychol. Assess..

[72] Park H.K., Chun S.Y., Choi Y., Lee S.Y., Kim S.J., Park E.-C. (2015). Effects of social activity on health-related quality of life according to age and gender: An observational study. Health Qual. Life Outcomes.

[73] Hawkley L.C., Cacioppo J.T. (2010). Loneliness Matters: A Theoretical and Empirical Review of Consequences and Mechanisms. Ann. Behav. Med..

[74] Pertz M., Okoniewski A., Schlegel U., Thoma P. (2020). Impairment of sociocognitive functions in patients with brain tumours. Neurosci. Biobehav. Rev..

[75] Pertz M., Popkirov S., Schlegel U., Thoma P. (2020). Research on cognitive and sociocognitive functions in patients with brain tumours: A bibliometric analysis and visualization of the scientific landscape. Neurol. Sci..

[76] Nakajima R., Kinoshita M., Nakada M., Herbet G. (2021). Social Cognition.

[77] Randazzo D., Peters K.B. (2016). Psychosocial distress and its effects on the health-related quality of life of primary brain tumor patients. CNS Oncol..

[78] Page M.J., McKenzie J.E., Bossuyt P.M., Boutron I., Hoffmann T.C., Mulrow C.D., Shamseer L., Tetzlaff J.M., Akl E.A., Brennan S.E. (2021). The PRISMA 2020 statement: An updated guideline for reporting systematic reviews. BMJ.

[79] Andrewes D.G., Kaye A., Murphy M., Harris B., Aitken S., Parr C., Bates L. (2003). Emotional and social dysfunction in patients following surgical treatment for brain tumour. J. Clin. Neurosci..

[80] Baird A., Dewar B.-K., Critchley H., Dolan R., Shallice T., Cipolotti L. (2006). Social and emotional functions in three patients with medial frontal lobe damage including the anterior cingulate cortex. Cogn. Neuropsychiatry.

[81] Baird A.D., Walker D.G., Biggs V., Robinson G.A. (2014). Selective preservation of the beat in apperceptive music agnosia: A case study. Cortex.

[82] Bowers D., Heilman K.M. (1984). Dissociation between the processing of affective and nonaffective faces: A case study. J. Clin. Neuropsychol..

[83] Bunston T., Mings D., Laperriere N., Malcolm J., Williams D. (1998). The impact of psychosocial need and needs resolution on quality of life in patients with brain tumors. FOC.

[84] Campanella F., Shallice T., Ius T., Fabbro F., Skrap M. (2014). Impact of brain tumour location on emotion and personality: A voxel-based lesion–symptom mapping study on mentalization processes. Brain.

[85] Campanella F., Fabbro F., Ius T., Shallice T., Skrap M. (2015). Acute effects of surgery on emotion and personality of brain tumor patients: Surgery impact, histological aspects, and recovery. Neuro-Oncology.

[86] Cavers D., Hacking B., Erridge S.E., Kendall M., Morris P.G., Murray S.A. (2012). Social, psychological and existential well-being in patients with glioma and their caregivers: A qualitative study. CMAJ.

[87] Channon S., Rule A., Maudgil D., Martinos M., Pellijeff A., Frankl J., Drury H., Shieff C. (2007). Interpretation of mentalistic actions and sarcastic remarks: Effects of frontal and posterior lesions on mentalising. Neuropsychologia.

[88] Chen P., Wang G., Ma R., Jing F., Zhang Y., Wang Y., Zhang P., Niu C., Zhang X. (2016). Multidimensional assessment of empathic abilities in patients with insular glioma. Cogn. Affect. Behav. Neurosci..

[89] Cornwell P., Dicks B., Fleming J., Haines T.P., Olson S. (2012). Care and support needs of patients and carers early post-discharge following treatment for non-malignant brain tumour: Establishing a new reality. Support. Care Cancer.

[90] Cubis L., Ownsworth T., Pinkham M.B., Foote M., Legg M., Chambers S. (2019). The importance of staying connected: Mediating and moderating effects of social group memberships on psychological well-being after brain tumor. Psychooncology.

[91] Giussani C., Pirillo D., Roux F.-E. (2010). Mirror of the soul: A cortical stimulation study on recognition of facial emotions. J. Neurosurg..

[92] Goebel S., von Harscher M., Mehdorn H.M. (2011). Comorbid mental disorders and psychosocial distress in patients with brain tumours and their spouses in the early treatment phase. Support. Care Cancer.

[93] Goebel S., Stark A.M., Kaup L., von Harscher M., Mehdorn H.M. (2011). Distress in patients with newly diagnosed brain tumours. Psychooncology.

[94] Goebel S., Mehdorn H.M., Wiesner C.D. (2018). Social cognition in patients with intracranial tumors: Do we forget something in the routine neuropsychological examination?. J. Neurooncol..

[95] Gu X., Gao Z., Wang X., Liu X., Knight R.T., Hof P.R., Fan J. (2012). Anterior insular cortex is necessary for empathetic pain perception. Brain.

[96] Guha-Thakurta N., Damek D., Pollack C., Hochberg F.H. (1999). Intravenous methotrexate as initial treatment for primary central nervous system lymphoma: Response to therapy and quality of life of patients. J. Neurooncol..

[97] Herbet G., Lafargue G., Bonnetblanc F., Moritz-Gasser S., Duffau H. (2013). Is the right frontal cortex really crucial in the mentalizing network? A longitudinal study in patients with a slow-growing lesion. Cortex.

[98] Herbet G., Lafargue G., Bonnetblanc F., Moritz-Gasser S., Menjot de Champfleur N., Duffau H. (2014). Inferring a dual-stream model of mentalizing from associative white matter fibres disconnection. Brain.

[99] Herbet G., Lafargue G., Moritz-Gasser S., Menjot de Champfleur N., Costi E., Bonnetblanc F., Duffau H. (2015). A disconnection account of subjective empathy impairments in diffuse low-grade glioma patients. Neuropsychologia.

[100] Herbet G., Lafargue G., Moritz-Gasser S., Bonnetblanc F., Duffau H. (2015). Interfering with the neural activity of mirror-related frontal areas impairs mentalistic inferences. Brain Struct. Funct..

[101] Jenkins L.M., Andrewes D.G., Nicholas C.L., Drummond K.J., Moffat B.A., Phal P., Desmond P., Kessels R.P.C. (2014). Social cognition in patients following surgery to the prefrontal cortex. Psychiatry Res. Neuroimaging.

[102] Kangas M., Williams J.R., Smee R.I. (2011). Benefit Finding in Adults Treated for Benign Meningioma Brain Tumours: Relations with Psychosocial Wellbeing. Brain Impair..

[103] Kangas M., Tate R.L., Williams J.R., Smee R.I. (2012). The effects of radiotherapy on psychosocial and cognitive functioning in adults with a primary brain tumor: A prospective evaluation. Neuro-Oncology.

[104] Kanter C., D’Agostino N.M., Daniels M., Stone A., Edelstein K. (2014). Together and apart: Providing psychosocial support for patients and families living with brain tumors. Support. Care Cancer.

[105] Langbecker D., Yates P. (2016). Primary brain tumor patients’ supportive care needs and multidisciplinary rehabilitation, community and psychosocial support services: Awareness, referral and utilization. J. Neurooncol..

[106] Lucas M.R. (2010). Psychosocial Implications for the Patient With a High-Grade Glioma. J. Neurosci. Nurs..

[107] Luherne-du Boullay V., Plaza M., Perrault A., Capelle L., Chaby L. (2014). Atypical crossmodal emotional integration in patients with gliomas. Brain Cogn..

[108] Mattavelli G., Pisoni A., Casarotti A., Comi A., Sera G., Riva M., Bizzi A., Rossi M., Bello L., Papagno C. (2017). Consequences of brain tumour resection on emotion recognition. J. Neuropsychol..

[109] Mu Y.-G., Huang L.-J., Li S.-Y., Ke C., Chen Y., Jin Y., Chen Z.-P. (2012). Working memory and the identification of facial expression in patients with left frontal glioma. Neuro-Oncology.

[110] Nakajima R., Kinoshita M., Okita H., Yahata T., Matsui M., Nakada M. (2018). Neural Networks Mediating High-Level Mentalizing in Patients with Right Cerebral Hemispheric Gliomas. Front. Behav. Neurosci..

[111] Nakajima R., Yordanova Y.N., Duffau H., Herbet G. (2018). Neuropsychological evidence for the crucial role of the right arcuate fasciculus in the face-based mentalizing network: A disconnection analysis. Neuropsychologia.

[112] Nakajima R., Kinoshita M., Okita H., Liu Z., Nakada M. (2021). Preserving Right Pre-motor and Posterior Prefrontal Cortices Contribute to Maintaining Overall Basic Emotion. Front. Hum. Neurosci..

[113] Ownsworth T., Chambers S., Hawkes A., Walker D.G., Shum D. (2011). Making sense of brain tumour: A qualitative investigation of personal and social processes of adjustment. Neuropsychol. Rehabil..

[114] Ownsworth T., Chambers S., Damborg E., Casey L., Walker D.G., Shum D.H.K. (2015). Evaluation of the making sense of brain tumor program: A randomized controlled trial of a home-based psychosocial intervention. Psycho-Oncology.

[115] Papagno C., Pisoni A., Mattavelli G., Casarotti A., Comi A., Fumagalli F., Vernice M., Fava E., Riva M., Bello L. (2016). Specific disgust processing in the left insula: New evidence from direct electrical stimulation. Neuropsychologia.

[116] Peper M., Irle E. (1997). Categorical and Dimensional Decoding of Emotional Intonations in Patients with Focal Brain Lesions. Brain Lang..

[117] Peper M., Irle E. (1997). The Decoding of Emotional Concepts in Patients with Focal Cerebral Lesions. Brain Cogn..

[118] Pertz M., Kowalski T., Thoma P., Schlegel U. (2021). What Is on Your Mind?: Impaired Social Cognition in Primary Central Nervous System Lymphoma Patients Despite Ongoing Complete Remission. Cancers.

[119] Prat-Acín R., Galeano-Senabre I., López-Ruiz P., Ayuso-Sacido A., Espert-Tortajada R. (2021). Intraoperative brain mapping of language, cognitive functions, and social cognition in awake surgery of low-grade gliomas located in the right non-dominant hemisphere. Clin. Neurol. Neurosurg..

[120] Saver J.L., Damasio A.R. (1991). Preserved access and processing of social knowledge in a patient with acquired sociopathy due to ventromedial frontal damage. Neuropsychologia.

[121] Shin J.Y., Kizilbash S.H., Robinson S.I., Uhm J.H., Hammack J.E., Lachance D.H., Buckner J.C., Jatoi A. (2016). Seizures in patients with primary brain tumors: What is their psychosocial impact?. J. Neurooncol..

[122] Sinha R., Dijkshoorn A.B.C., Li C., Manly T., Price S.J. (2020). Glioblastoma surgery related emotion recognition deficits are associated with right cerebral hemisphere tract changes. Brain Commun..

[123] Szelag E., Fersten E. (1991). Recognition of faces expressing emotions in patients with unilateral brain damage. Acta Neurobiol. Exp..

[124] Trejnowska A., Goodall K., Rush R., Ellison M., McVittie C. (2020). The relationship between adult attachment and coping with brain tumour: The mediating role of social support. Psycho-Oncology.

[125] Troschel F.M., Ahndorf F., Wille L.-M., Brandt R., Jost J., Rekowski S., Eich H.T., Stummer W., Wiewrodt R., Jetschke K. (2021). Quality of Life in Brain Tumor Patients and Their Relatives Heavily Depends on Social Support Factors during the COVID-19 Pandemic. Cancers.

[126] Voß H., Scholz-Kreisel P., Richter C., Ringel F., Singer S., Renovanz M. (2021). Development of screening questions for doctor–patient consultation assessing the quality of life and psychosocial burden of glioma patients: An explorative study. Qual. Life Res..

[127] Wang X., Gu X., Fan J., Wang S., Zhao F., Hof P.R., Liu P., Gao Z. (2014). Recovery of empathetic function following resection of insular gliomas. J. Neurooncol..

[128] Weitzner M.A., Meyers C.A., Byrne K. (1996). Psychosocial functioning and quality of life in patients with primary brain tumors. J. Neurosurg..

[129] Yordanova Y.N., Duffau H., Herbet G. (2017). Neural pathways subserving face-based mentalizing. Brain Struct. Funct..

[130] Yuksek E., Eroz S., Yassa A., Akturk D., Zakirov F., Akcam F.E., Emul M. (2015). The Influences of Whole Brain Radiotherapy on Social Cognition and Association with Hippocampal and Frontal Dosimetry. Psychiatr. Q..

[131] (2018). The cooperative human. Nat. Hum. Behav..

[132] Arnold S.D., Forman L.M., Brigidi B.D., Carter K.E., Schweitzer H.A., Quinn H.E., Guill A.B., Herndon J.E., Raynor R.H. (2008). Evaluation and characterization of generalized anxiety and depression in patients with primary brain tumors. Neuro-Oncology.

[133] Manne S., Badr H. (2008). Intimacy and relationship processes in couples’ psychosocial adaptation to cancer. Cancer.

[134] Doolittle N.D., Korfel A., Lubow M.A., Schorb E., Schlegel U., Rogowski S., Fu R., Dósa E., Illerhaus G., Kraemer D.F. (2013). Long-term cognitive function, neuroimaging, and quality of life in primary CNS lymphoma. Neurology.

[135] Day J., Gillespie D.C., Rooney A.G., Bulbeck H.J., Zienius K., Boele F., Grant R. (2016). Neurocognitive Deficits and Neurocognitive Rehabilitation in Adult Brain Tumors. Curr. Treat. Options Neurol..

[136] Noll K.R., Bradshaw M.E., Weinberg J.S., Wefel J.S. (2017). Relationships between neurocognitive functioning, mood, and quality of life in patients with temporal lobe glioma. Psychooncology.

[137] Litofsky N.S., Farace E., Anderson F., Meyers C.A., Huang W., Laws E.R. (2004). Depression in patients with high-grade glioma: Results of the Glioma Outcomes Project. Neurosurgery.

[138] Henry J.D., von Hippel W., Molenberghs P., Lee T., Sachdev P.S. (2016). Clinical assessment of social cognitive function in neurological disorders. Nat. Rev. Neurol..

[139] Burgess P., Alderman N., Forbes C., Costello A., Coates L.M., Dawson D., Anderson N., Gilbert S., Dumontheil I., Channon S. (2006). The case for the development and use of “ecologically valid” measures of executive function in experimental and clinical neuropsychology. J. Int. Neuropsychol. Soc..

[140] Njomboro P. (2017). Social Cognition Deficits: Current Position and Future Directions for Neuropsychological Interventions in Cerebrovascular Disease. Behav. Neurol..

[141] Giovagnoli A.R. (2012). Investigation of cognitive impairments in people with brain tumors. J. Neurooncol..

[142] Wallis K., Kelly M., McRae S.E., Mcdonald S., Campbell L.E. (2021). Domains and measures of social cognition in acquired brain injury: A scoping review. Neuropsychol. Rehabil..

[143] Shamay-Tsoory S.G. (2011). The Neural Bases for Empathy. Neuroscientist.

[144] Channon S. (2004). Frontal lobe dysfunction and everyday problem-solving: Social and non-social contributions. Acta Psychol..

[145] Rowe A.D., Bullock P.R., Polkey C.E., Morris R.G. (2001). ‘Theory of mind’ impairments and their relationship to executive functioning following frontal lobe excisions. Brain.

[146] Fanning J.R., Bell M.D., Fiszdon J.M. (2012). Is it possible to have impaired neurocognition but good social cognition in schizophrenia?. Schizophr. Res..

[147] Lagravinese G., Avanzino L., Raffo De Ferrari A., Marchese R., Serrati C., Mandich P., Abbruzzese G., Pelosin E. (2017). Theory of Mind Is Impaired in Mild to Moderate Huntington’s Disease Independently from Global Cognitive Functioning. Front. Psychol..

[148] Meyers C.A., Wefel J.S. (2003). The Use of the Mini-Mental State Examination to Assess Cognitive Functioning in Cancer Trials: No Ifs, Ands, Buts, or Sensitivity. J. Clin. Oncol..

[149] Kroenke C.H., Kubzansky L.D., Schernhammer E.S., Holmes M.D., Kawachi I. (2006). Social networks, social support, and survival after breast cancer diagnosis. J. Clin. Oncol..

[150] Lutgendorf S.K., de Geest K., Bender D., Ahmed A., Goodheart M.J., Dahmoush L., Zimmerman M.B., Penedo F.J., Lucci J.A., Ganjei-Azar P. (2012). Social influences on clinical outcomes of patients with ovarian cancer. J. Clin. Oncol..

[151] Fox S., Lantz C. (1998). The Brain Tumor Experience and Quality of Life: A Qualitative Study. J. Neurosci. Nurs..

[152] Janda M., Eakin E.G., Bailey L., Walker D., Troy K. (2006). Supportive care needs of people with brain tumours and their carers. Support. Care Cancer.

[153] Courtens A.M., Stevens F.C.J., Crebolder H.F.J.M., Philipsen H. (1996). Longitudinal study on quality of life and social support in cancer patients. Cancer Nurs..

[154] McConigley R., Halkett G., Lobb E., Nowak A. (2010). Caring for someone with high-grade glioma: A time of rapid change for caregivers. Palliat. Med..

[155] Schmer C., Ward-Smith P., Latham S., Salacz M. (2008). When a family member has a malignant brain tumor: The caregiver perspective. J. Neurosci. Nurs..

[156] Schubart J.R., Kinzie M.B., Farace E. (2008). Caring for the brain tumor patient: Family caregiver burden and unmet needs. Neuro-Oncology.

[157] Fiszdon J.M., Reddy L.F. (2012). Review of social cognitive treatments for psychosis. Clin. Psychol. Rev..

[158] Vallat-Azouvi C., Azouvi P., Le-Bornec G., Brunet-Gouet E. (2018). Treatment of social cognition impairments in patients with traumatic brain injury: A critical review. Brain Inj..

[159] Cassel A., McDonald S., Kelly M. (2020). Establishing ‘proof of concept’ for a social cognition group treatment program (SIFT IT) after traumatic brain injury: Two case studies. Brain Inj..

[160] Kurtz M.M., Richardson C.L. (2012). Social Cognitive Training for Schizophrenia: A Meta-Analytic Investigation of Controlled Research. Schizophr. Bull..

[161] Combs D.R., Adams S.D., Penn D.L., Roberts D., Tiegreen J., Stem P. (2007). Social Cognition and Interaction Training (SCIT) for inpatients with schizophrenia spectrum disorders: Preliminary findings. Schizophr. Res..

[162] Bornhofen C., Mcdonald S. (2008). Treating deficits in emotion perception following traumatic brain injury. Neuropsychol. Rehabil..

[163] Bucher J.A., Loscalzo M., Zabora J., Houts P.S., BrintzenhofeSzoc K. (2001). Problem-solving cancer care education for patients and caregivers. Cancer Pract..

[164] Dinapoli L., Chiesa S., Dinapoli N., Gatta R., Beghella Bartoli F., Bracci S., Mazzarella C., Sanfilippo M.Z., Sabatino G., Gaudino S. (2021). Personalised support of brain tumour patients during radiotherapy based on psychological profile and quality of life. Support. Care Cancer.

[165] Penton-Voak I.S., Munafò M.R., Looi C.Y. (2017). Biased Facial-Emotion Perception in Mental Health Disorders: A Possible Target for Psychological Intervention?. Curr. Dir. Psychol. Sci..

