# Supplementary material for: Sociocognitive Functioning and Psychosocial Burden in Patients with Brain Tumors
Source: Cancers (Basel). 2022 Feb 1;14(3):767. doi: 10.3390/cancers14030767 (PMC8833643; doi:10.3390/cancers14030767)
Supplement: Supplementary file 1 [file cancers-14-00767-s001.zip › cancers-1523418-supplementary.pdf]

Table S1. Summary of the time of assessment, number of patients, control group and matching criteria as well as key findings concerning socio-cognitive functioning or psychosocial burden of the identified studies.

Authors	Time of Assessment [1]	Number of Patients with Diagnosis	Control Group [2]	Matching Criteria with the Control Group	Key Findings/Research Objective Concerning Sociocognitive Functioning or Psychosocial Burden
Andrewes et al. (2003)	outpatient clinic; 6-12 or 12-18 months after surgery	self-ratings of the emotional and social dysfunction questionnaire available for: n = 26 meningioma; n = 13 neuroma; n = 11 astrocytoma; n = 17 pituitary adenoma; partner-ratings of the emotional and social dysfunction questionnaire available for: n = 21 meningioma; n = 12 neuroma; n = 14 astrocytoma; n = 13 pituitary adenoma	yes	31 patients after surgery to extra-cerebral areas and 18 terminally ill cancer patients; 49 controls (self-ratings); n = 44 controls (partner-ratings); matching for age and education	• more emotional and social dysfunction in patients following tumor surgery as compared to controls<br>• astrocytoma patients: more problems on self-ratings and partner version of the questionnaire compared to patients diagnosed with meningioma<br>• malignant tumor patients were rated worse on the partner- and self-rated version of the questionnaire compared to benign tumor patients<br>• tumor classification and location of lesions made separate contributions on emotional profile
Baird et al. (2006)	after treatment	patient 1: removal of right prefrontal glioma with unilateral anterior cingulate cortex lesion; patient 2: partial resection of an oligodendroglioma with bilateral involvement of anterior cingulate cortex and broader parts of the brain	yes	emotional expression multimorph task: 14 age and education-matched healthy controls; joke interpretation task: 8 age and education-matched healthy controls; social situations and ToM tasks: 15 age and education-matched healthy controls	• emotional facial expression multimorph task: patient 1 did not significantly differ from controls across all emotions, but non-significant trend for the patient to require more stages to recognize fear, happiness and surprise than controls<br>• patient 2: more errors than controls in recognizing surprise and non-significant trend for the patient to require more stages than controls to recognize surprise<br>• social situation task: no significant differences between patients and healthy controls, but trend for the patients´ judgement of appropriateness to be less severe as compared to controls<br>• joke interpretation: performance of patient 1 was comparable to controls, performance of patient 2 was poor (tended to use physical interpretations rather than mental state interpretations)<br>• advanced ToM task: performance of patient 1 was equal with controls, performance of patient 2 was impaired (significantly higher number of physical justifications and fewer mental state justifications)

Baird et al. (2014)	2 months after surgery	1 patient after resection of right temporoparietal WHO grade II oligodendroglioma	yes	5 age-, gender-, education and musical experienced-matched controls	• patient performed significantly below controls in identifying sad and peaceful music excerpts<br>• for happy and scary excerpts the performance was comparable to controls<br>• Awareness of Social Inferences Test: 11/12 correct, only when identifying anxious and revolted emotions the patient scored at the 5 % cut-off and made 2 false positive responses for surprise
Bowers & Heilman (1984)	1 day after chemotherapy has begun; evaluation throughout the next week	1 patient with a tumor of the deep white matter of the occipito-temporo-parietal region of the right hemisphere	yes	comparison to data from right- and left-hemispheric patients previously evaluated	• Neutral Facial Discrimination Task: 100 % correct<br>• Name the Facial Emotion Task: 56 % correct<br>• Chose the Facial Emotion Task: 60 % correct<br>• Same-Different Facial Emotion Task: 100 % correct<br>• Affective Prosody Task: 94 % correct
Bunston et al. (1998)	after treatment; outpatient setting; time since diagnosis on average 35.4 months; 2 assessments (1 week apart) only to verify reliability and validity of inventory	75 outpatients with primary brain tumors: n = 25 astrocytoma; n = 14 glioblastoma; n = 10 oligodendroglioma; n = 5 medulloblastoma; n = 5 mixed glioma; n = 3 lymphoma; n = 12 other; n = 1 tumor type not reported	no	-	• 97.3 % of patients reported at least one concern<br>• average number of needs was 22.4 out of possible 58<br>• on average patients had needs in more than half of the 12 domains<br>• need concerning healthcare system (53.3 %), treatment side effects (84 %), maintenance of activities of daily living (54.7 %), controlling uncertainty (82.7 %), self and body image (66.7 %), nutrition (46.7 %), finances and employment (60 %), family (62.7 %), belonging (36 %), social support (41.3 %), emotional distress (58.7 %) and meaningful existence (73.3 %)<br>• no association between demographic characteristics and patients´ QoL<br>• all need domains associated with QoL<br>• the greater the number of needs the lower QoL (e.g. the need for social support (r = .34) and family (r = .55) was associated with QoL)
Campanella et al. (2014)	a few days before (usually the day before) surgery and a few days (usually within a	71 brain tumor patients: n = 29 high-grade glioma; n = 21 low-grade glioma; n = 16 meningioma; n = 5 metastasis	mention of control sample scores without further descrip-	not described	• greater difficulties in identifying facial emotions for temporal patients (who obtained average scores below the control´s cut off) with respect to parietal patients (who obtained average scores in line with controls)<br>• emotion recognition: 16/20 of the temporal patients performed worse than controls either before or after surgery (or both)<br>• temporal lobe patients had significantly more difficulties in identifying fear than parietal patients<br>• parietal patients were equally good in identifying all emotions (apart from

	week) after surgery		tion		an advantage of happiness over anger and surprise)<br>• frontal patients: happiness was easier to identify than all other emotions<br>• temporal patients: happiness was easier to identify than all other emotions, except for disgust that was better recognized than fear and anger<br>• frontal and temporal groups: significantly lower RMET scores compared to parietal patients (no differences between frontal and temporal patient groups)<br>• RMET: 10/20 of temporal lobe patients performed worse than 1 SD below the reference populations as did 10/28 of the frontal patients either before or after surgery or both<br>• the frontal group showed significantly higher alexithymia scores than both other groups<br>• Temperament and Character Inventory: no significant effects at group level
Campanella et al. (2015)	before (usually the day before), immediately after (usually within the week after) and for low-grade glioma patients 4 months after surgery (14/21)	66 brain tumor patients: n = 29 high-grade glioma; n = 21 low-grade glioma; n = 16 meningioma	yes	20 healthy controls matched for age and education for emotion recognition; normative values for the other tasks	• emotion recognition and ToM: maximally affected by temporal lesions<br>• alexithymia and Assessment of Character: maximally affected by frontal lesions<br>• high-grade glioma patients: more difficulties in emotion recognition and ToM with respect to patients with meningiomas<br>• low-grade glioma patients: no difficulty in emotion recognition in general compared with meningioma patients<br>• both high-grade glioma and meningioma patients were unaffected by surgery and maintained the level of performance after surgery as before on emotion recognition and ToM<br>• low-grade glioma participants worsened in their performance on emotion recognition (clinically relevant scores 9.5 % versus 42.1 %) and ToM (clinically relevant scores 5.3 % versus 33.3 %) after surgery<br>• no effect of surgery for alexithymia and Temperament and Character Inventory<br>• 4 months after surgery low-grade glioma significantly recovered their emotional recognition and ToM with respect to immediate post-surgical performance<br>• alexithymia: low-grade glioma patients showed a mild but consistent reduction of scores with respect to both before and immediately after surgery

Cavers et al. (2012)	before pathological diagnosis (suspected glioma); after diagnosis and immediately preceding treatment with chemotherapy or radiotherapy; after treatment ended; at 6 months follow-up; post bereavement	26 patients undergoing investigation for glioma or with confirmed diagnosis: n = 2 astrocytoma; n = 15 glioblastoma; n = 1 oligodendroglioma; n = 1 brain stem glioma; n = 2 anaplastic astrocytoma; n = 5 not a glioma); 21 patients with confirmed glioma diagnosis participated in further interviews; 23 patients´ relatives	no	-	• social trajectory/lives of patients substantially disrupted at time of diagnosis<br>• patients´ time was occupied with in-patient stays, visits to hospital and recovery from surgery leaving little time to invest in social well-being<br>• patients have a strong desire to return to "normal" socially after treatment<br>• for some patients the effort of socializing posed a barrier to returning to life as normal<br>• as disease progressed the sense of isolation increased<br>• during the terminal phase of the illness: people´s social lives dwindled alongside their physical and cognitive abilities
Channon et al. (2007).	post-operative, before any radiotherapy	n = 23 patients with frontal brain tumors; n = 22 patients with posterior brain tumors; n = 26 glioma; n = 12 meningioma; n = 3 metastasis; n = 1 hemangiopericytoma; n = 1 neurocytoma; n = 1 abscess; n = 1 CVA	yes	26 healthy controls matched for age and years of education or intelligence	• no significant impairments for non-mentalistic control questions<br>• generation of free responses: frontal group scored below healthy controls for actions, direct sarcasm and indirect sarcasm<br>• selection of alternatives: frontal group scored below the healthy controls for actions, direct sarcasm, indirect sarcasm concerning correct alternatives<br>• patients with frontal lesions: impaired performance for both free responses and selection of alternatives on each set of mentalistic material (intact comprehension of the control non-mentalistic items)
Chen et al. (2016)	before surgery and therapy; within 1 week after seizure onset	34 patients with localized glioma limited to the insular (n = 17) or a posterior non-insular area (n = 17)	yes	30 right-handed healthy controls; matching for age, gender, education and ethnicity	• alexithymia: lower scores for insular glioma patients<br>• patients with an insular glioma: significantly different from the other patient group on the empathic concern and perspective taking subscales of IRI<br>• insular patients: impaired in cognitive empathy and affective empathy, as compared to both the healthy controls and patients with posterior gliomas<br>• insula patients were significantly different from the other 2 groups in emotion recognition, perception of others´ pain and emotional perspective taking concerning correct responses<br>• reactions times: only differences for perception of others´ pain task with the

					insular and non-insular lesion patients were significantly different from healthy controls<br>• no obvious laterality effects on affective and cognitive empathy (right versus left insula)
Cornwell et al. (2012)	2 weeks post-discharge from hospital; 3 months post-discharge from hospital	9 patients with non-malignant brain tumors: n = 5 meningioma; n = 1 atypical choroid plexus papilloma; n = 2 pituitary macroadenoma; n = 1 pituitary adenoma; all patients had undergone neurosurgical excision and none had received radiotherapy or chemotherapy during study period	no	-	• 3 primary themes: coping with available support, adjusting to routines and relationships, emotional responses<br>• second primary themes: adjustment to daily routines and lifestyle changes for patients on return home following discharge<br>• challenges faced in relation to daily tasks and activities patients usually were capable of performing prior to surgery<br>• change in household roles after discharge from hospital often increased the burden for carers and was accompanied by both a sense of new dependence and appreciation by the patient<br>• patients were aware of possible adjustments regarding their vocational roles post-surgery<br>• impact on personal relationships: positive and negative changes throughout recovery processes<br>• difficulties with day-to-day communication and understanding between patients and others in their household<br>• emotions distress accompanied by acute feelings of loneliness<br>• experience of isolation from others, regardless of having available people to contact
Cubis et al. (2019)	after treatment; at mean 61.7 months since diagnosis	70 primary brain tumor patients: n = 32 benign; n = 13 low-grade glioma; n = 25 high-grade glioma	no	-	• older age related to greater maintenance of social group membership and fewer new social group memberships<br>• objective global cognitive status not significantly associated with perceived physical impairment, social group membership or psychological well being<br>• greater perceived physical impairment significantly associated with lower maintenance or loss of social group memberships, lover confidence in social group membership and fewer new social group memberships<br>• greater perceived cognitive impairment significantly associated with the loss of social group memberships but not with confidence in social group membership<br>• loss of social group membership significantly associated with higher levels of depression, anxiety and lower life satisfaction<br>• lower confidence in social group membership associated with higher depression and anxiety and lower life satisfaction

Giussani et al. (2010)	intraoperative cortical stimulation; neuropsychological examination before and after surgery	18 patients with right hemispheric lesions: n = 5 metastases; n = 6 high-grade glioma; n = 4 low-grade glioma; n = 2 arteriovenous malformations; n = 1 malignant meningioma	no	-	• none of the patients had a significant postoperative facial emotion recognition deficit<br>• postoperative scores of facial emotion recognition similar to preoperative scores in 16 patients<br>• scores were slightly lower than the preoperative score in 2 patients
Goebel et al. (2011)	after first neurosurgical treatment within first 3 months after brain tumor detection (n = 19 < 1 months since diagnosis; n = 7 1-3 months since diagnosis); testing within 5-14 days after surgery; no chemotherapy or radiotherapy at the time of testing	26 brain tumor patients: n = 12 meningioma; n = 4 astrocytoma; n = 5 glioblastoma; n = 5 other	no	-	• prevalence of comorbid mental disorders based on the clinical interview: 38 % for patients<br>• acute stress disorder: most prominent diagnosis in the patient group with 19 %<br>• 73 % of patients described relevant psychosocial stress<br>• 77 % of patients experienced the first diagnosis of a brain tumor as distressing followed by the fear of surgery side-effects (65 %)
Goebel et al. (2011)	5-14 days after surgery	159 patients with solitary primary intracranial tumors: n = 67 astrocytoma; n = 41 glioblastoma; n = 2 gliosarcoma; n = 18 diffuse astrocytoma WHO grade II-III; n = 3 oligodendro-	no	-	• mean distress score: 5.51 (SD = 2.86)<br>• cut-off of 6: 48.4 % classified as significantly distressed<br>• on average 6.86 items of concern in the problem list<br>• majority of problems: physical, followed by emotional, practical, familial, and spiritual problems<br>• distress scores significantly positively correlated with patient-reported

		glioma WHO grade II-III; n = 2 oligoastrocytoma grade WHO II-III; n = 1 ganglioglioma WHO grade I; n = 62 meningioma; n = 13 schwannoma; n = 11 pituitary adenoma; n = 6 others			overall number of problems<br>• problems of dealing with the partner or with children were reported by 5 % of patients respectively<br>• distress and number of problems associated with anxiety, depression and social support
Goebel et al. (2018)	preoperative	30 patients with solitary intracranial tumors: n = 9 glioblastoma; n =8 astrocytoma WHO grade I-III; n = 4 meningioma; n = 4 oligodendroglioma, n = 5 other	yes	healthy controls matched for sex, age and education	• 25 patients (83 %) impaired in at least one measure of social cognition<br>• significant differences between patients and healthy controls for facial differentiation, emotion recognition, ToM, complex ToM reasoning, nonverbal affective ToM<br>• patients with a more malignant tumor: greater difficulties in emotion recognition<br>• no differences between patients with left and right-sided tumors<br>• patients with frontal tumors: greatest impairments in the Faux-Pas Test<br>• patients with temporal tumors: greatest impairments in Faux-Pas Test, emotion discrimination, emotion recognition, the picture stories cognitive ToM and by trend in the RMET<br>• mean participation score of cognitive and social participation (Marburg Competence Scale): 31.95 (range 19-51)<br>• mean score of social integration on the social adjustment scale: 1.66 (range 1-3.03)<br>• patients with more severe difficulties in emotion discrimination described less emotional burden in the HADS<br>• significant association between social and occupational functional assessment and cognitive ToM<br>• Faux-Pas Test: most sensitive in discriminating between patients and controls<br>• combination of Faux-Pas Test and RMET: 77 % diagnosed with social cognitive impairment
Gu et al. (2012)	after surgery	low-grade glioma patients: 3 with focal unilateral anterior insular cortex lesions; 3 with focal dorsal anterior cingulate cortex lesions	yes	brain-damaged controls: 6 patients with focal lesions in regions other than the anterior insular or	• patients with anterior insular cortex lesions: significant diminished ability to discriminate painful from non-painful stimuli as compared to both control groups<br>• patients with anterior cingulate cortex lesions: no impairments in discriminating painful from non-painful stimuli<br>• anterior insular cortex lesions, but not anterior cingulate cortex lesions dis-

				anterior cingulate cortices (e.g. lateral prefrontal cortex and temporal pole); 14 neurologically intact participants; matching for age, education and ethnicity	rupted the typical interference effect of empathy for pain on discriminability of laterality<br>• discrete anterior insular cortex lesions, but not anterior cingulate cortex lesions, result in deficits in explicit and implicit pain perception
Guha-Thakurta et al. (1999)	after initial treatment with intravenous methotrexate without radiotherapy; median duration of complete remission: 16 months; median follow-up since diagnosis: 22 months	11 patients with PCNSL	modified FACT-Brain: 26 post-operative malignant glioma patients of a phase II drug trail; Symptom Questionnaire: normative data from another study ("community	normative data used for the different questionnaires; not further described	• FACT mean total score: 158.6 in PCNSL patients and 134.9 in 26 glioma patients (total score and subscale scores of "symptoms" and "memory" significantly different)<br>• PCNSL patients scored non-significant better for subscales of energy, leisure, cognition, socializing, work and sexuality<br>• higher scores for depression and anxiety but also for somatic well-being and lower for anger in 9 patients included in the symptomatic questionnaire in comparison to the normal population<br>• comparable performance of 9 PCNSL patients on the Social Adjustment Scale Self-Report to the community for work, social-leisure, extended family interactions, marital functions and family-unit functions but less for parental functions<br>• comparable estimates of problem solving abilities and stress coping abilities in the Problem Solving Inventory between PCNSL patients and the normal population

sample"
n = 50
people
without
known
malig-
nancy or
neuro-
logic
disease);
the So-
cial
Ad-
justment
Scale
Self-Rep
ort:
norma-
tive data
from a
"com-
munity
sample
of n =
399
people;
the
Problem
Solving
Inven-
tory:
norma-
tive data
from a
"com-
munity

			sample" of n = 900 individuals		
Herbet et al. (2013)	the day before, 5 days after and 3 months after surgery	10 patients with WHO grade II glioma invading at least a substantial part of the right frontal lobe	yes	10 healthy subjects matched for age, socio-educational level and manual laterality; no history of neurologic/psychiatric disorder or drug/alcohol abuse	• patient and control group: no preoperative differences on RMET<br>• significant difference on RMET immediately postoperative but not 3 months postoperative<br>• although non-significant group differences at months 3 for RMET, incomplete functional recovery (especially if inferior frontal gyrus pars opercularis was resected)<br>• dorsomedial prefrontal group: increase in reaction times in the attribution of intention condition of the Comic Strip Task but only transiently (impairment fully resolved by month 3)<br>• dorsomedial prefrontal group: more errors, specifically in the attribution of intention condition compared to other groups (fronto-temporo-insular lesioned patients and control group)<br>• widespread surgical excisions of the right prefrontal cortex without long-term worsening of sociocognitive performance<br>• transitory effects immediately after surgery
Herbet et al. (2014)	at least 3 months after surgery	93 patients after resection of diffuse low-grade glioma	yes	60 healthy subjects: RMET (n = 42) and Comic Strip Task (n = 18)	• RMET mean and SD: 61.67 ± 11 % correct responses in patient group<br>• Comic Strip Task mean and SD: 84.69 ± 13.34 % of correct responses in the attribution of intentions condition and 93.75 ± 7.29 % of correct responses in the physical causality condition in patient group
Herbet et al. (2015)	after surgery	107 patients after surgery for diffuse low-grade glioma	no, but comparison to validation studies and French and English norms	-	• mean ± SD scores in patient sample: 38.94 ± 10.08 (females) and 33.25 ± 9.57 (males)<br>• mean scores for both males and females were inferior to those in previous validation studies<br>• compared to French norms: 29.99 % of patients had low empathy scores, 10.18 % showed abnormally low scores, 10.28 % had high empathy scores, 4.76 % showed abnormally high scores

Herbet et al. (2015)	RMET the day before surgery; intraoperative RMET during electrical stimulation	5 patients with a diffuse low-grade right-hemisphere glioma	yes	24 healthy controls (from 2 recent studies) used to transform the patients´ scores into age adjusted z-scores	• the day before surgery: normal performance levels for all patients compared to healthy controls
Jenkins et al. (2014)	1 to 12 months after surgery	27 patients following prefrontal cortex surgery: n = 9 grade I tumors; n = 5 grade II tumors; n = 4 grade III tumors; n = 3 grade IV tumors; n = 2 single metastatic tumor; n = 1 angioma; n = 1 epidermoid cyst; n = 1 abscess; n = 1 bone fragment	yes	26 patients post-spinal surgery (cervical and lumbar laminectomy); serious neurosurgical operation without cerebral involvement	• Emotion recognition Task: ventromedial group scored significantly lower than controls<br>• ventromedial lesion location: significant predictor of identification accuracy of facial emotional recognition<br>• ventromedial group performed significantly worse than the controls for fear<br>• Perspective Taking Task: no significant differences between brain tumor and control groups on the physical scale<br>• both ventromedial and dorsolateral prefrontal group performed worse than control group on the ToM scale<br>• dorsolateral prefrontal group scored significantly lower than controls on empathy scale<br>• ventromedial lesion: significant predictor of poor ToM
Kangas et al. (2011)	on average 4.4 years after diagnosis (2 months - 22.6 years); 1.5 years after completion of radiation (1 - 9.26 years)	70 meningioma patients: early subgroup with diagnosis within the past 2 year period (n = 27) and late subgroup (n = 43) diagnosis for more than 2 years	no	-	• perceived social support: no groups differences between early and late subgroup<br>• early subgroup: more anxiety/tension and more anger/hostility symptoms than the late subgroup<br>• one of the most commonly endorsed items: valuing the social support received from established/existing networks<br>• high sample mean for perceived social support (M = 73.0, SD = 9.6)
Kangas et al. (2012)	before (15.22 ± 17.6 days) and 3.5 months after radiotherapy	primary brain tumor patients: n = 23 with left-hemispheric benign tumors; n = 22 with right-hemispheric benign tumors; n = 11 with	no	-	• benign tumor patients: substantially elevated post-traumatic stress symptoms and general distress at baseline relative to patients with a malignant tumor<br>• benign subgroup: significant reduction in post-traumatic stress symptoms and improvement in emotional well-being postradiotherapy, especially for

	(3.54 ± 0.84 months)	left-hemispheric malignant tumors; n = 11 with right-hemispheric malignant tumors			individuals with left-hemispheric tumors<br>• malignant brain tumor patients: significant reduction in social well-being and heightened social constraints in their interpersonal relationships postradiotherapy relative to benign brain tumor patients<br>• individuals with a left-hemispheric malignancy: substantially lower social well-being at follow-up compared to patients with right-hemispheric malignancy<br>• 44 % of patients: post-traumatic stress symptoms before radiation<br>• 56 % of patients: chronic post-traumatic stress<br>• high level of post-traumatic stress associated with reduced QoL and poor perceived quality of social support both pre- and postradiotherapy<br>• fatigue: most prevalent symptom experienced at both time points
Kanter et al. (2014)	after treatment; time since diagnosis 1.08 ± 1.98 months (attended a support group once) and 1.21 ± 1.86 months (attended more than once)	137 brain tumors patients; resources of medical charts for 80 patients: n = 36 glioblastoma; n = 26 low-grade glioma; n = 11 anaplastic glioma; n = 7 other (meningioma, PCNSL, acoustic schwannoma, plasmacytoma, primitive neuroectodermal tumor)	no	-	• 7 themes from patient support groups identified, with "relationships" being one of the themes<br>• balancing the need to receive care and provide it to others<br>• role reversals, strain and concerns about the impact of illness on caregivers<br>• losing the ability to parent<br>• common topics: impact of illness on intimacy and sexuality, concerns about social relationships
Langbecker & Yates (2016)	immediately after recruitment and approximately 3 months later; all assessments within the first 6 months after	40 newly diagnosed primary brain tumor patients: n = 16 glioblastoma; n = 9 meningioma; n = 3 astrocytoma; n = 2 oligodendroglioma; n = 10 other	no	-	• highest mean supportive care needs score at each time point for physical needs, closely followed by psychological needs<br>• mean scores in all domains except for sexuality decreased over time<br>• psychosocial needs at baseline mean 45.8 (25.1) and at follow up mean 35.6 (27.5)

	diagnosis				
Lucas (2010)	outpatient clinic; not further specified	brain tumor patients with a high-grade glioma (WHO grade III or IV); number not further specified	no	-	• 3 themes of experiences<br>• one theme: loss of relationships<br>• mood fluctuations and irritability caused by tumor location, treatment, medication, and/or frustration most frequently influences exchanges with those closest in proximity and closest in emotional attachment
Luherne-du Boulay et al. (2014)	before oncological treatment	26 primary brain tumor patients: n = 1 oligodendroglioma grade III; n = 10 mixed glioma grade II; n = 6 oligodendroglioma grade II; n = 2 astrocytoma grade II; n = 2 glioblastoma; n = 4 mixed glioma grade III; n = 1 mixed glioma grade IV	yes	26 healthy controls without neurological and psychiatric illness; matching for age and gender	• accuracy scores on emotion recognition: patient group performed overall more poorly than healthy controls<br>• patients performed more poorly than healthy controls on the visual and auditory modality<br>• no group difference concerning crossmodal emotion recognition<br>• reaction times: patients were consistently slower than controls<br>• deficits were minor (mean accuracy of 87.82 % for faces and 87.95 % for voices)<br>• efficient compensatory mechanisms during tumor development
Mattavelli et al. (2017)	before and after surgery (± 7 days); 3 months after surgery in subgroup of patients	34 patients: n = 7 glioblastoma; n = 3 oligodendroglioma; n = 5 astrocytoma; n = 8 anaplastic astrocytoma; n = 1 gliosarcoma; n = 2 ganglioglioma; n = 1 brain metastasis; n = 1 anaplastic oligodendroglioma; n = 6 oligoastrocytoma	yes	17 healthy controls matched by age and educational level	• pre-surgery: left-hemispheric patients scored significantly lower than controls on fearful facial expressions<br>• facial emotion recognition task: left-hemispheric patients' performance decreased for all emotions but fear, which was poorly recognized already before surgery<br>• right-hemispheric patients: only sadness and fear facial recognition decreased after tumor resection<br>• no overall impairment in auditory emotion recognition before surgery<br>• auditory emotion recognition task: patients' performance decreased after surgery without significant difference between right- and left-hemispheric lesions<br>• follow-up: general good recovery at 3 months after surgery
Mu et al. (2012)	3-5 days before surgery	11 brain tumor patients: n = 2 astrocytoma grade II; n = 2 glioblastoma; n = 3 anaplastic astrocytoma; n = 2 astrocytoma grade I; n = 2 oligodendroglioma grade II	yes	11 healthy volunteers individually matched by age, sex and education	• correct identified number of expressions: non-significantly better in control group than in patients<br>• correct recognition rate of angry faces: significantly lower among patients than among control subjects<br>• patients were significantly less accurate in detecting angry faces than were control subjects (30.3 % versus 57.6 %)<br>• no deficits in the identification of other expressions

Nakajima et al. (2018)	before surgery, 1 week after surgery, 3 months after surgery; assessment during awake surgery in 8 patients ("with intraoperative mapping group")	20 patients with right-hemispheric glioma: n = 5 diffuse astrocytoma; n = 3 oligodendroglioma; n = 3 anaplastic astrocytoma; n = 4 anaplastic oligodendroglioma; n = 5 glioblastoma	yes	18 normal healthy volunteers matched for age and MMSE score	• lower accuracy in patients than in healthy controls before and 1 week after surgery<br>• no significant difference between the score of all patients and healthy controls at 3 months after surgery<br>• lower scores of both the with and without intraoperative mapping groups than of healthy volunteers at preoperative and postoperative 1 week<br>• 3 months postoperative: normalization of scores only in the group with intra-operative mapping
Nakajima et al. (2018)	at least 3 months after surgery	122 patients after resection of diffuse low-grade glioma	no	-	• raw total RMET score: 23.7 ± 3.8 (13-33)<br>• 13.9 % of patients with (significant) deficit<br>• raw total scores on average 23.9 ± 3.5 (right tumor, n = 92) and 23.1 ± 4.5 (left tumor, n = 30)<br>• number of patients assigned to the ‘impaired’ subgroup: 22/92 in right lesion group, 7/30 in left lesion group
Nakajima et al. (2021)	preoperative, 1 week postoperative and until 3 months after surgery; intraoperative mapping	22 patients with right hemispheric glioma with successful intraoperative assessment of basic emotion: n = 2 dysembryoplastic neuroepithelial tumors; n = 2 diffuse astrocytoma; n = 4 oligodendroglioma; n = 1 ependymoma; n = 2 anaplastic astrocytoma; n = 7 anaplastic oligodendroglioma; n = 4 glioblastoma	age-matched controls for z-scores; group characteristics not specified	not specified	• After surgery: basic emotion test score decreased in 9 patients compared to preoperative score, of these, it decreased under the cut-off value (z ≤ 1.65) in 3 patients<br>• total score decreased significantly just after surgery (preoperative: 0.21 ± 0.61; postoperative 1 week: - 0.98 ± 1.15)<br>• recovery to preoperative level within 3 months postoperatively (- 0.28 ± 0.78)
Ownsworth et al. (2011)	between 4 months and 21 years after diagnosis; all	18 brain tumor patients: n = 3 meningioma; n = 2 low-grade astrocytoma; n = 1 low-grade oligodendroglioma; n = 1 col-	no	-	• primary theme: “key sense-making appraisals”<br>• secondary themes: “interactions with those in the healthcare system”, “reactions and support from people in the support network” and “diversity of coping efforts”

	patients had treatment with surgery and either radiation or chemotherapy or both	loid cyst; n = 1 pituitary macroadenoma; n = 1 craniopharyngioma; n = 4 glioblastoma; n = 3 anaplastic oligodendroglioma; n = 1 anaplastic astrocytoma; n = 1 multiple brain metastases			• many participants experienced strain in their relationships due to the illness and changes in functioning<br>• behavioral changes related to the tumor: particularly serious effects on relationships<br>• some participants attributed the loss of a relationship to the tumor, particularly those in a new relationship<br>• loss of relationships or strain placed on these as a source of distress and disappointment<br>• other patients: more positive effects on relationships (illness had brought them closer)
Ownsworth et al. (2015)	after medical treatment: surgery (78 %) with adjuvant treatment of chemotherapy (52 %) and/or radiation therapy (42 %); before, after and 6 months after psychosocial program	50 brain tumor patients: n = 15 glioblastoma; n = 7 oligodendroglioma; n = 7 astrocytoma; n = 6 meningioma; n = 6 pituitary tumor, n = 2 colloid cyst; n = 2 craniopharyngioma	yes; 25/27 of the immediate group completed the "making sense of a brain tumour program"; 19/23 of the wait-list group completed the program; n = 44 completed the pro-	wait-list and control group matched for age, education, gender, time since diagnosis, tumor location, malignancy and global neuropsychological status	• significantly lower levels of depression and higher existential well-being, functional well-being and QoL at post-treatment after intervention compared to wait-list controls<br>• no significant between-group differences at post-assessment on social/family well-being, emotional well-being, overall QoL, depression, anxiety and stress (potential explanation: only 60 % had a family member involved in therapy)<br>• relative to pre-intervention, at 6 months follow-up participants reported significantly lower levels of depression, anxiety and stress and significantly higher levels of existential well-being and overall QoL (all participants who received the psychosocial intervention)<br>• psychosocial intervention: improving existential and functional well-being and reducing depressive symptoms

			gram and post-assessment (n = 25 immediate, n = 19 waitlist); n = 36 completed 6 months follow-up		
Papagno et al. (2016)	the day before and the week after surgery; intraoperative mapping	13 brain tumor patients with a preoperative performance on the modified Ekman test of at least 80 %: n = 1 focal cortical dysplasia IIa; n = 2 oligodendroglioma grade II; n = 3 anaplastic oligoastrocytoma grade III; n = 3 anaplastic astrocytoma grade III; n = 2 glioblastoma; n = 1 metastasis; n = 1 oligoastrocytoma grade II; 1 patient with right-hemisphere tumor not included in analyses	yes	12 neurologically unimpaired controls; matching for age and years of education	• percentage of correct responses: 80 % in the non-stimulation condition<br>• percentage of correct responses 59 % in the stimulation condition (mainly involved the antero-superior part of the insula and to a lesser extent the posterior-superior cortex)<br>• significant difference between stimulated and non-stimulated trials only for disgust<br>• no effects of stimulation on remaining emotions<br>• accuracy post-surgery significantly lower compared to pre-surgery performance (88 % versus 78 %)<br>• overall emotion recognition performance decreased after surgery without specific effects for single emotions
Peper & Irle (1997)	in most cases approximately 8 days post-operatively	41 brain tumor patients affecting the cerebral cortex: n = 16 meningiomas; n = 4 solitary metastases; n = 6 astrocytoma grade I-II; n = 11 astrocytoma grade III-IV; n = 3 others (exclusion of 1 left-handed patient from analyses)	yes	12 patients with surgery for slipped discs or peripheral nerve disease; matching for age, sex and education	• all lesion groups: significantly reduced selection of emotion labels as compared to controls<br>• marginally significant effect for hemisphere: lower performance of the right-hemispheric group as compared to controls<br>• right-hemispheric patients were impaired in recognizing all categories except for joy<br>• left-hemispheric patients differed from controls only in the recognition of sad vocalizations

					• marginal significant effect for localization: slightly lower dimensional decoding in patients with left or right posterior lesions<br>• trend of lower valence decoding in left-hemispheric compared to right-hemispheric patients and controls<br>• left-hemispheric patients decode negative valence trials with lesser accuracy as compared to right-hemispheric patients and controls<br>• right-hemispheric patients: decoding of both high and low arousal items was impaired in comparison to controls<br>• crossmodal categorial recognition: impaired in right-hemispheric patients (left-hemispheric patients´ performance comparable to controls)<br>• dimensional decoding task: reduced recognition of valence in left-hemispheric and arousal in right-hemispheric patients
Peper & Irle (1997)	at median 8 days postoperatively; inclusion of some preoperative data due to missing postoperative assessment	70 brain tumor patients affecting the cerebral cortex after microsurgical tumor extirpation: n = 23 meningioma; n = 6 solitary metastases; n = 9 astrocytoma grade I-II; n = 25 astrocytoma grade III-IV; n = 6 others; exclusion of 1 left-handed patient	yes	15 patients after surgery for slipped discs or peripheral nerve disease; matching for age, sex, education	• dimensional decoding: impaired decoding of arousal in all groups relative to controls, except for the left frontal group; right parietal group was most severely impaired, when compared to the left frontal group<br>• component of negative valence: reduced in all lesion groups in comparison to controls, with the right frontal group and the right parietal group being clearly affected<br>• categorial decoding: right parietal and left temporal group showed an impaired performance as compared to controls<br>• discrimination of emotion categories: lower performance for right parietal lesioned patients as compared to controls (group was particularly impaired in discriminating the category of anger)
Pertz et al. (2021)	at median 35 months (12-98) after complete remission to therapy	43 PCNSL patient	yes	43 healthy controls; matching for age, gender, years of school and years of education	• decreased behavioral cognitive empathy in PCNSL patients as compared to healthy controls (irrespective of valence)<br>• no differences on control questions of the Social Problem Solving Task for PCNSL patients and healthy controls<br>• PCNSL patients rated the degree of discomfort of an awkward situation on a comparable level as healthy controls<br>• decreased ability to accurately identify the element that evokes awkwardness in PCNSL patients<br>• both the production and the mere recognition of optimal solutions for interpersonal conflicts were impaired in PCNSL patients in the Social Problem Solving Fluency task

Prat-Acin et al. (2021)	before surgery and 6 months after surgery; intraoperative mapping task	observational cohort: 15 right-hemispheric diffuse low-grade glioma (WHO grade II astrocytoma) patients with intraoperative cortical and subcortical brain mapping during an asleep-awake-asleep surgery	yes	control cohort: 15 right-hemispheric diffuse low-grade glioma (WHO grade II astrocytoma) patients operated under general anesthesia; matching for tumor location and histology	• partial stimulations: higher number of incorrect responses in social cognition task<br>• neuropsychological assessment at 6 months: 1 patient worsened in the observational group compared to preoperative baseline (non-social control task: decrease in naming task and semantic task)<br>• 6 patients presented with decreased performance at 6 months in the control group in at least 1 task (3 patients in naming and semantic task, 2 patients in working memory and attention and 1 patient in the social cognition task)
Saver & Damasio (1991)	after surgery	1 meningioma patient after bilateral surgery of ventromedial frontal cortices	yes	5 subjects; matching for sex, age, intelligence, education and occupation	• patient performs at levels equivalent or superior to normal controls on diverse components of social cognition<br>• normal ability to generate a wide variety of response options to problematic social situations<br>• tendency to consider spontaneously the consequences of particular social response options<br>• capacity to conceptualize effective measures towards achieving given social objectives<br>• ability to predict the likely outcome of a particular social situation<br>• sharp contrast between intact functioning in laboratory tasks and profoundly deficient social decision-making in real life
Shin et al. (2016)	after multimodality therapy: n = 27 surgery; n = 25 radiation; n = 25 chemotherapy	27 malignant primary brain tumor patients with a history of 1 or more seizures: n = 9 glioblastoma; n = 7 anaplastic astrocytoma; n = 9 oligodendroglioma; n = 2 low-grade astrocytoma	no	-	• patients described how the seizures led to a loss of independence associated with an inability to take care of children, to work, to drive, to do hobbies or to be alone<br>• heavy unmitigated psychosocial burden
Sinha et al. (2020)	before surgery (at median 3 days), after surgery (at median 3 days)	15 glioblastoma patients	normative data	mean and SD for emotion recognition task, but group assessment not further specified	• longitudinal difference in emotion recognition<br>• decrease after surgery (before surgery: 3/15 patient with a deficit in emotion recognition; after surgery 7/15 patients with an emotion recognition deficit)

Szelag & Fersten (1991)	3 months - 12 years after surgery	12 brain tumor patients (classified as mild and removed completely): n = 2 astrocytoma; n = 3 intracerebral hematoma; n = 1 sarcoma; n = 6 meningioma	yes	18 persons without damage to the central nervous system	• different pattern of asymmetry in patients with right hemispheric damage compared to both the control group and patients with left hemisphere damage<br>• happy faces: level of errors in left and right visual field similar in the control group, mean percent of errors in the right visual field was lower than that observed in the left visual field in patients with right-hemispheric damage<br>• sad faces: fewer recognition errors for expositions in the left than in the right visual field in patients with left hemispheric damage, in patients with right hemispheric damage fewer errors for the recognition of faces in the right than in the left visual field<br>• neutral faces: level of errors in the left visual field was lower than in the right one in controls and patients with left hemispheric damage, patients with a right hemispheric damage made fewer errors in the right visual field than in the left one
Trejnowska et al. (2020)	illness duration at median 34 months; stage of illness via self-report: n = 13 recently diagnosed; n = 84 undergoing active treatment; n = 337 stable disease or "watch and wait"; n = 21 disease progressing; n =25 no further treatment possible	480 primary brain tumor patients: n = 166 meningioma; n = 77 astrocytoma other than glioblastoma; n = 59 oligodendroglioma; n = 57 glioblastoma; n = 15 acoustic neuroma; n = 10 pituitary adenoma; n = 9 ependymoma; n = 9 craniopharyngioma; n = 8 epidermoid; n = 53 other types; n = 17 unsure	no	-	• mean value of social support: 3.68 (SD = 1.00)<br>• individual´s attachment representations have implications for the coping strategies<br>• association of high levels of attachment anxiety and helplessness/hopelessness and anxious preoccupation strategies<br>• association of high levels of attachment avoidance and helplessness/hopelessness<br>• perceived social support partially explained the relationship between both attachment dimensions and helplessness/hopelessness<br>• association of attachment anxiety with anxious preoccupation not mediated by social support<br>• neither attachment nor social support associated with the cognitive avoidance style

Troschel et al. (2021)	weekly over a period of 12 weeks during the first COVID-19 pandemic lockdown between 22 April and 15 July 2020	63 brain tumor patients: n = 5 meningioma; n = 23 astrocytoma; n = 20 glioblastoma; n = 8 oligodendroglioma; n = 3 neurinoma; n = 3 plexus papilloma; n = 2 ependymoma; n = 2 solitary fibrous tumor; n = 1 germinoma; n = 1 ganglioglioma	comparison with relatives of the patients (n = 37)	-	• patients: higher HADS Depression score across all time points and lower WHO5 score when compared to relatives<br>• 113/484 patient submitted questionnaires (23.3 %) and 24/245 (9.8 %) of relative submitted questionnaires met the cutoff of 10 for elevated depression<br>• Anxiety: 114/484 (23.5 %) versus 40/245 (16.3 %) met threshold<br>• QoL: stable over the period of 12 weeks<br>• patients and relatives tended to miss social contacts less at the conclusion of the study period when compared to the beginning<br>• most consistent predictor of QoL: number of social contacts<br>• 10 or more social contacts associated with a 70 % reduced risk for more depression symptoms, a 39 % reduced risk for more anxiety symptoms, and a 65 % reduced risk for increased distress while enhancing the chance of increased well-being by 73 % when compared to 0-3 social contacts per week<br>• QoL strongly improved with increased social contacts
Voß et al. (2021)	62 (0.7-369) months after diagnosis	50 patients: n = 23 glioblastoma, WHO grade IV, IDH wildtype; n = 3 astrocytoma, WHO grade III, IDH wildtype; n = 12 astrocytoma, WHO grade III, IDH mutated; n = 2 astrocytoma, WHO grade II, IDH mutated; n = 3 anaplastic oligoastrocytoma, NOS; n = 5 oligodendroglioma, WHO grade III, IDH mutated; n = 2 ependymoma WHO grade II	no	-	• topics psyche, body and cognition considered to be most relevant for patients<br>• in weighted scoring procedure: psyche (18.9); cognition (22.9), body (21.4); role functioning (18.3); social support (17.2); unmet needs (15.5)
Wang et al. (2014)	before and 3 months after surgery	4 patients with unilateral insular gliomas WHO grade II or III: n = 2 astrocytoma; n = 1 oligodendroglioma; n = 1 anaplastic oligodendroglioma	yes	18 neurologically intact individuals; matching for age, gender, education and ethnicity	• diminished ability to discriminate painful from non-painful stimuli in preoperative insular glioma patients<br>• sensitivity to others pain was significantly improved in insula patients postoperatively<br>• no significant differences between patients and healthy controls neither pre- nor postoperative concerning reaction times<br>• implicit sensitivity to pain was impaired preoperative and restored in insular patients postoperatively<br>• no differences concerning IRI or Toronto Alexithymia Scale

Weitzner et al. (1996)	after treatment; on average 30.7 months since diagnosis (1-154)	50 primary brain tumor patients: 60 % anaplastic astrocytoma; 30 % glioblastoma; 10 % other primary brain tumors; after surgical resection adjunctive treatment with chemotherapy (91 %) and radiotherapy (78 %)	no	-	• QoL affected adversely by 1 or more of 5 factors: being female, being divorced, having bilateral tumor involvement, having received chemotherapy and showing a poor performance status
Yordanova et al. (2017)	the day before, 5 days and 3 months after surgery; direct electrical stimulation during surgery	27 right-hemispheric diffuse low-grade glioma patients	yes	behavioral data from 54 healthy controls from 2 other studies used to transform the preoperative patients´ individual scores into z-scores; matching for age, educational level and sex ratio	• before surgery: only 1 patient had a deficit<br>• strong performance decrease right after surgery (preoperative versus immediate postoperative)<br>• no significant difference between performance before surgery and 3 months after surgery
Yuksek et al. (2015)	before and 1 month after radiotherapy	13 primary or secondary brain tumor patients: n = 2 postoperative glioblastoma; n = 1 postoperative anaplastic ependymoma; n = 2 postoperative anaplastic oligodendroglioma; n = 2 meningioma; n = 3 solitary brain metastasis from lung adenocarcinoma; n = 3 small cell lung cancer for prophylactic whole brain radiotherapy	yes	13 healthy controls; matching for age and gender	• patients after radiotherapy: significantly better rate of recognizing fear than before radiation<br>• trend towards higher accuracy in identifying sadness and surprise after radiation as compared to before<br>• patients after radiation: significantly better in recognizing fear and significantly impaired in recognizing angry and neutral facial emotions compared to controls<br>• no significant differences concerning response times

Note.
[1] pre- or post-treatment
[2] clinical or healthy control group
Cerebrovascular Accident (CVA)
Hospital Anxiety and Depression Scale HADS

Interpersonal Reactivity Index (IRI)
Mini Mental State Examination (MMSE)
Primary Central Nervous System Lymphoma (PCNSL)
Quality of Life (QoL)
Reading the Mind in the Eyes Test (RMET)
Standard Deviations (SD)
The Functional Assessment of Cancer Therapy (FACT)
Theory of Mind (ToM)
World Health Organization (WHO)
